# Chemotherapy-Free Targeted Anti-BCR-ABL+ Acute Lymphoblastic Leukemia Therapy May Benefit the Heart

**DOI:** 10.3390/cancers14040983

**Published:** 2022-02-15

**Authors:** Hanna Kirchhoff, Melanie Ricke-Hoch, Katharina Wohlan, Stefan Pietzsch, Ümran Karsli, Sergej Erschow, Robert Zweigerdt, Arnold Ganser, Matthias Eder, Michaela Scherr, Denise Hilfiker-Kleiner

**Affiliations:** 1 Department of Hematology, Hemostasis, Oncology and Stem Cell Transplantation, Hannover Medical School, 30625 Hannover, Germany; kirchhoff.hanna@mh-hannover.de (H.K.); katharina.wohlan@bcm.edu (K.W.); karsli.uemran@mh-hannover.de (Ü.K.); ganser.arnold@mh-hannover.de (A.G.); scherr.michaela@mh-hannover.de (M.S.); 2 Department of Cardiology and Angiology, Hannover Medical School, 30625 Hannover, Germany; pietzsch.stefan@mh-hannover.de (S.P.); erschow.sergej@mh-hannover.de (S.E.); hilfiker.denise@mh-hannover.de (D.H.-K.); 3 Department of Molecular and Cellular Biology, Baylor College of Medicine, Houston, TX 77030, USA; 4 Department of Human Genetics, Hannover Medical School, 30625 Hannover, Germany; 5 Department of Gastroenterology, Hepatology & Endocrinology, Hannover Medical School, 30625 Hannover, Germany; 6 Leibniz Research Laboratories for Biotechnology and Artificial Organs (LEBAO), Hannover Medical School, 30625 Hannover, Germany; zweigerdt.robert@mh-hannover.de; 7 Department of Cardiovascular Complications of Oncologic Therapies, Medical Faculty of the Philipps University Marburg, 35037 Marburg, Germany

**Keywords:** cardio-oncology, cardiovascular disease, cancer, acute lymphoblastic leukemia, targeted anti-cancer therapies

## Abstract

**Simple Summary:**

Risk-adapted multiagent chemotherapy has led to a remarkable improvement in the life expectancy of patients with acute lymphoblastic leukemia (ALL). Nevertheless, in high-risk subgroups such as BCR-ABL+ ALL, relapse rates remain high without allogeneic hematopoietic stem cell transplantation, and the adverse effects of chemotherapy may cause acute and chronic cardiac complications or dysfunction. Here, we demonstrated that chemotherapy-free targeted therapies designed to optimize apoptosis induction in BCR-ABL+ ALL may circumvent cardiac on-target side effects and may even activate cardiac recovery.

**Abstract:**

Targeted therapies are currently considered the best cost–benefit anti-cancer treatment. In hematological malignancies, however, relapse rates and non-hematopoietic side effects including cardiotoxicity remain high. Here, we describe significant heart damage due to advanced acute lymphoblastic leukemia (ALL) with t(9;22) encoding the bcr-abl oncogene (BCR-ABL+ ALL) in murine xenotransplantation models. Echocardiography reveals severe cardiac dysfunction with impaired left ventricular function and reduced heart and cardiomyocyte dimensions associated with increased apoptosis. This cardiac damage is fully reversible, but cardiac recovery depends on the therapy used to induce ALL remission. Chemotherapy-free combination therapy with dasatinib (DAS), venetoclax (VEN) (targeting the BCR-ABL oncoprotein and mitochondrial B-cell CLL/Lymphoma 2 (BCL2), respectively), and dexamethasone (DEX) can fully revert cardiac defects, whereas the depletion of otherwise identical ALL in a genetic model using herpes simplex virus type 1 thymidine kinase (HSV-TK) cannot. Mechanistically, dexamethasone induces a pro-apoptotic BCL2-interacting mediator of cell death (BIM) expression and apoptosis in ALL cells but enhances pro-survival B-cell lymphoma extra-large (BCLXL) expression in cardiomyocytes and clinical recovery with the reversion of cardiac atrophy. These data demonstrate that therapies designed to optimize apoptosis induction in ALL may circumvent cardiac on-target side effects and may even activate cardiac recovery. In the future, combining the careful clinical monitoring of cardiotoxicity in leukemic patients with the further characterization of organ-specific side effects and signaling pathways activated by malignancy and/or anti-tumor therapies seems reasonable.

## 1. Introduction

Acute lymphoblastic leukemia (ALL) is a heterogeneous cancer disease of lymphoid blood cells, characterized by the accumulation of large numbers of immature lymphocytes, often harboring multiple genetic aberrations. The Philadelphia translocation t(9;22)(q34;q11) defines a very high-risk subtype of B cell precursor (BCP) ALL [1,2,3]. The resulting chimeric fusion protein BCR-ABL has constitutive tyrosine kinase activity that is essential for malignant transformation. The targeted inhibition of BCR-ABL tyrosine kinase activity by tyrosine kinase inhibitors (TKI), such as dasatinib (DAS) induction and post-remission chemotherapy regimens, has markedly improved patient outcomes for this high-risk subgroup [3,4]. Nevertheless, without allogeneic hematopoietic stem cell transplantation, relapse rates remain high [5,6] and the adverse effects of chemotherapy may cause cardiac dysfunction. Therefore, the combination of TKI with known cardiotoxic anti-cancer drugs such as anthracyclines is not recommended.

The specific and direct induction of mitochondrial-mediated apoptosis by BCL2 homology domain 3 (BH3) mimetics such as venetoclax (VEN) has been demonstrated to be especially effective in lymphoid malignancies [7,8]. VEN competitively binds to anti-apoptotic B-cell CLL/Lymphoma 2 (BCL2), and therefore disrupts its binding to pro-apoptotic proteins of the BCL2-like protein family (mainly BCL2-interacting mediator of cell death (BIM)). This induces activation of the effector proteins, BCL2-associated X (BAX) and BCL2-antagonist/Killer (BAK) and culminates in mitochondrial outer-membrane permeabilization (MOMP), the point of no return in intrinsic apoptosis induction [7]. Its efficacy can be potentiated by suitable combination therapy approaches [9,10,11].

Current targeted therapies combining the specific inhibition of the BCR-ABL oncoprotein by TKIs such as DAS with conventional polychemotherapy are currently considered the best treatment for BCR-ABL+ ALL. Accordingly, the curative potential of DAS, VEN and dexamethasone (DEX) combination therapy has been evaluated in preclinical murine xenotransplantation models for high-risk BCR-ABL+ ALL with regard to clinically relevant endpoints [9,10]. However, targeted anti-tumor therapies may exert unexpected on- and off-target side effects in organ systems that are not affected by malignant transformation or malignant cells. For example, cardiovascular toxicities have been described for trastuzumab, bevacizumab, imatinib and sunitinib, which were widely used for therapy for solid tumors and chronic myeloid leukemia (CML), respectively [12,13,14]. Therefore, we hypothesized that murine xenograft models may be used to both optimize anti-leukemic therapy and characterize and prevent side effects on the heart where applicable.

The aim of this study was to analyze the impact of BCR-ABL+ ALL on the heart, as well as the effect of an anti-cancer pharmacotherapy consisting of DAS, DEX and VEN in terms of function and molecular mechanisms. To date, VEN has not been reported to induce severe cardiovascular problems. Nevertheless, the combination of multiple anti-cancer agents can cause unexpected side effects or minimize therapeutic windows. TKIs such as DAS are known to cause several cardiac complications such as congestive heart failure and arrhythmia and uncommon cardiac adverse events including angina pectoris, cardiomegaly, pericarditis, ventricular arrhythmia and myocardial infarction [15,16], and should therefore be closely monitored with regard to cardiac side effects. 

## 2. Materials and Methods

Cell culture media were obtained from Biochrom (Berlin, Germany) and Invitrogen (Waltham, MA, USA). All other chemicals, except the pharmacologic agents listed below, were purchased from Sigma-Aldrich (St. Louis, MO, USA).

### 2.1. Pharmacologic Agents

For in vitro experiments, VEN (SelleckChem, Houston, TX, USA) and DAS (Santa Cruz Biotechnology, Dallas, TX, USA) were solubilized in dimethyl sulfoxide (DMSO) (Merck, Darmstadt, Germany) to 10 mM stocks. For in vivo studies, VEN and DAS were solubilized in a vehicle consisting of 60% Phosal, 30% PEG 400 and 10% Ethanol to 20 mg/mL and 40 mg/mL, respectively. DEX (SelleckChem, Houston, TX, USA) was solubilized in phosphate buffered saline (PBS) at stock concentrations of 10 mM (in vitro) and 20 mg/mL (in vivo). Ganciclovir (GCV, HEXAL, Holzkirchen, Germany) was stored in stock solution of 50 mg/mL in 0.9% NaCL.

### 2.2. Patient Samples

Primary BCR-ABL+ ALL cells were obtained with written informed consent and in accordance with the declaration of Helsinki. Cells were isolated via ficoll density gradient and subsequently stored in liquid nitrogen. For co-culture experiments, cells were thawed in SFEM II medium (Stemcell Technologies, Cambridge, UK) supplemented with 20% fetal calf serum (FCS), 1% penicillin/streptomycin (P/S) (Gibco, Grand Island, NY, USA), 20 ng/mL interleukin (IL)-3 and 10 ng/mL IL-7 (both Peprotech, Hamburg, Germany).

### 2.3. Cell Lines

The BCR-ABL-positive B-cell leukemia cell line BV173 (DSMZ, Braunschweig, Germany) was cultivated in RPMI1640 supplemented with 10% FCS and 1% P/S.

### 2.4. Preparation of Lentiviral Supernatants and Transduction

The preparations of recombinant lentiviral supernatants and lentiviral transductions were performed as described earlier [17]. Transduction efficacy was assessed by enhanced green fluorescent protein (eGFP), or yellow fluorescent protein (YFP), expression using a FACS Calibur with CellQuest Pro software 4.0.2 (BD Bioscience, San Jose, CA, USA).

### 2.5. Isolation and Culture of Primary Adult Mouse Cardiomyocytes

Cardiomyocytes (CM) were isolated by enzymatic digestion from C57BL/6N (Charles River, Sulzfeld, Germany) as previously described, and subsequently plated on laminin-coated tissue culture plates from a subset of mice [18]. Isolated CM were cultivated in 1× MEM culture medium (Gibco, Grand Island, NY, USA) supplemented with 1% penicillin, 1% L-glutamine, and 25 mM (−)-blebbistatin (Cayman Chem, Ann Arbor, MI, USA) in 1% CO_2_ at 37 °C in a cell culture incubator and were treated with VEN (100 nM), DAS (150 nM) and DEX (500 nM) alone or in combination for 24 h.

### 2.6. Culture and Cardiomyogenic Differentiation of Human Induced Pluripotent Stem Cells (iPSCs)

Human iPSCs were grown in mTeSR or E8 medium (Stemcell Technologies, Cambridge, UK) as described [19]. In brief, cells were passaged every 3–4 days using Accutase (Life Technologies, Carlsbad, CA, USA) and reseeded at 0.5 × 10^4^ cells/mL on Geltrex-coated (Life Technologies, Carlsbad, CA, USA) culture flasks including 10 mM rho kinase (ROCK) inhibitor (Y-27632, Stemcell Technologies, Vancouver, BC, Canada). For the directed, chemically defined differentiation, single cells were inoculated in E8 for pre-culture using Erlenmeyer flasks (20 mL working volume; rotated at 70 rpm on an orbital shaker) for aggregate formation in suspension [20]. The cell density was determined after 48 h and adjusted to 5 × 10^5^ cells/mL for differentiation in CDM3 medium supplemented with 5 mM CHIR (SelleckChem, Houston, TX, USA) and 5 mM Y-27632, as described [21]. Precisely 24 h later, the medium was replaced by CDM3 supplemented with a 5 mM inhibitor of Wnt production 2 (IWP2) (ReproCell, Betsville, MD, USA); 48 h later, the medium was replaced with pure CDM3; fresh CDM3 medium was added every 2–3 days thereafter. The resulting iPSC cardiomyocytes (iPSC-CM) were analyzed on day 10 of differentiation, as reported [21], and used for experiments within 1–2 weeks. Cells were treated with VEN (100 nM), DAS (150 nM) and DEX (500 nM) alone or in combination for 24 h, and total RNA was isolated using Trizol (Life Technologies, Carlsbad, CA, USA).

### 2.7. Isolation and Culture of Human Mesenchymal Stem Cells (MSCs) from Bone Marrow

For studies involving human bone marrow, ethical approval was obtained from the ethical committee of Hannover Medical School. Samples were collected in accordance with the Declaration of Helsinki after written informed consent was received from the respective donors. Bone marrow samples were obtained during resection of the femoral head for implantation of a total hip arthroplasty in otherwise healthy donors. Human MSCs were isolated from these samples by density gradient centrifugation and subsequent plastic adhesion of mononuclear cells and cultured as described [22]. MSC preparations were routinely frozen in passage 2 with 95 % (*v/v*) FCS/5 % (*v/v*) DMSO and stored at −140 °C.

### 2.8. Co-Culture of ALL Cells on MSCs

MSCs were seeded at a density of 4000 cells/well in a 96-well plate in MSC medium 24 h prior to adding B-ALL cells. B-ALL cells were seeded onto MSCs at a density of 1 × 10^6^ cells/mL in SFEM II medium (Stemcell Technologies, Cambridge, UK) supplemented with 20% FCS, 20 ng/mL recombinant IL3 and 10 ng/mL recombinant IL7 (both Peprotech, Hamburg, Germany). After at least 4 h of co-culture, VEN, DAS and DEX were added in combination or as single agents in a concentration range from 1–1000 nM in fixed ratios. After 48 h of drug exposure, non-adherent cells present in supernatant medium were collected, followed by trypsinization and collection of the adherent cell fraction, which contains both MSCs and adhered ALL cells. Cell suspension was stained with human anti-CD19-APC antibody (Biolegend, San Diego, CA, USA) for 20 min at RT and subsequently stained with 1 µg/mL propidium iodide (PI) (Serva, Heidelberg, Germany). Cell viability of CD19-positive cells was assessed via flow cytometry in a FACS Calibur and analyzed with CellQuest Pro software 4.0.2 (both BD Bioscience, San Jose, CA, USA). Relative cell viability was normalized to untreated controls. Drug synergy was analyzed using CompuSyn software 1.0 (BioSoft, Cambridge, UK).

### 2.9. Animal Experiments

All animal studies were in accordance with the German animal protection law and with the European Communities Council Directive 86/609/EEC and 2010/63/EU for the protection of animals used for experimental purposes. All experiments were approved by the Local Institutional Animal Care and Research Advisory Committee and permitted by the local authority, the Niedersächsisches Landesamt für Verbraucherschutz und Lebensmittelsicherheit (No. 33.14-42502-04-16/2217), (33.12.42502-04-17/2520) and (No. 33.19-42502-05-18A271).

Leukemic BV173 cells or patient-derived xenograft (PDX) cells (1 × 10^6^) were intravenously transplanted into recipient female NSG (NOD.Cg-Prkdcscid Il2rgtm1WjI/SzJ) mice (10 ± 2 weeks of age) and, after cell implantation, mice received continuous analgesia (Novalgin, 1000 mg/kg/day in drinking water; HEXAL, Holzkirchen, Germany) and antibiosis (0.08 mg/mL in drinking water Ciprofloxacin) as described [9]. The combination of DAS (10 mg/kg) and DEX was administered by oral gavage in 0.1M sodium citrate 5 days per week; VEN (20 mg/kg) was applied orally with a treatment delay of minimum 2 h in a vehicle consisting of 60% Phosal, 30% PEG 400 and 10% Ethanol as described [9]. The health condition of animals was assessed based on the guidelines of recognition and distress in experimental animals, as proposed by Morton and Griffiths [23] and mice were euthanized when endpoint criteria were met. Where possible, data were analyzed by an experimenter blinded to the tumor status and treatment regimen of the animals.

### 2.10. In Vivo Bioluminescence Imaging

BV173 and PDX (L4951) cells were transduced with lentiviral vectors SLIEW (or HSV-TK) encoding both enhanced GFP (or YFP), for in vitro analysis, and firefly luciferase, for in vivo bioluminescence imaging (BLI) [18,24,25]. Tumor burden was assessed by BLI using an in vivo imaging system (IVIS) Lumina II (Caliper Life Sciences, Hopkinton, MA, USA) [9,10,18]. D-Luciferin (0.8 mg/mouse) (AppliChem, Darmstadt, Germany) was provided intraperitoneally and whole-animal imaging was performed from a ventral perspective. Bioluminescence radiance was analyzed using Living Image 4.0 software.

### 2.11. Echocardiography

Transthoracic 2D echocardiography was performed to assess the heart rate (HR) and contractile function in NSG female leukemia-bearing mice (BV173 18w, PDX 28w) and in corresponding healthy age-matched controls (1–4% isoflurane inhalation, connected to a rodent ventilator) using a linear 20–46 Mhz transducer (Vevo 770 or MX400, Vevo 3100, Visual Sonics, Toronto, ON, Canada) as described [18]. Parasternal long-axis (PSLAX) views were recorded in B-mode (LV trace B-mode for Vevo 3100) at the level of the papillary muscle, and still images were used to measure left ventricular (LV) end-diastolic diameter (LVEDD) and LV end-systolic diameter (LVESD), end-diastolic area (EDA), end-systolic area (ESA) and calculate fractional area change (FAC) (end-diastolic area—end-systolic area/end-diastolic area × 100). Cardiac output (LV CO) calculated as (stroke volume × (heart rate at the first frame drawn/1000)), endocardial stroke volume (ESV; calculates as diastolic—systolic endocardial volume), endocardial diastolic volume (EVd; ((7.0/(2.4 + LVEDD)) × LVEDD^3^), and endocardial systolic volume (EVs; ((7.0/(2.4 + LVESD)) × LVESD^3^)) were calculated from the automatic tracing by Visual Sonics Vevo 770 software version 3 and Vevo lab 2.1.0 software (Visual Sonics, Toronto, ON, Canada)

### 2.12. Histology and Immunostaining

For cardiac morphological analyses, hearts were embedded in OCT Tissue-Tek and frozen at −80 °C or were fixed in formalin and embedded in paraffin as described [18]. Cardiomyocyte cross-sectional area (CSA) was determined on longitudinal 6 μm LV cryo-sections after rhodamine-labelled wheat germ agglutinin (WGA, RL-1022, Vector Laboratories, Burlingame, CA, USA)/Hoechst 33258 staining (SIGMA-Aldrich, St. Louis, MO, USA), as previously described [18]. At least 30–60 representative cardiomyocytes (3 sections/heart) were measured per heart.

For histological analyses of apoptosis, hearts were fixed in formalin and embedded in paraffin as described [26]. Apoptotic nuclei were detected by in situ terminal deoxynucleotidyl transferase-mediated digoxigenin-conjugated dUTP nick end labeling (TUNEL) using the ApopTag^®^ fluorescein in situ apoptosis detection kit (EMD Millipore, Billerica, MA, USA) according to the manufacturer’s protocol on longitudinal paraffin heart sections and by nuclear morphology using Hoechst 33258 staining [26]. Sections were co-stained with rhodamine-labelled WGA. The average number of apoptotic cells was determined per section (3 sections/animal) and the area of each section was measured for the calculation of apoptotic cells/area.

Images and subsequent measurements of CSA and TUNEL were taken by fluorescence microscopy with an Axiovert 200 M microscope using AxioVision 4.8 software or Axio Observer 7 using Zen 2.6 software (Carl Zeiss, Jena, Germany).

### 2.13. Immunoblotting

Whole-cell lysates of BV173 were prepared with lysis buffer (20 mM HEPES, pH 7.5, 0.4 M NaCl; 1 mM EDTA, 1 mM EGTA, 1 mM DTT) supplemented with mini complete protease inhibitor cocktail tablet (Roche Diagnostics, Mannheim, Germany) [9]. LV tissue was lysed as described previously [18]. Protein lysates were separated by sodium dodecyl sulphate-polyacrylamide gel electrophoresis (SDS-PAGE), transferred to Hybond-enhanced chemiluminescence (ECL) nitrocellulose membrane (Amersham Bioscience, Uppsala, Sweden) and membranes were incubated with the following antibodies according to the manufacturer’s protocol: anti-BCLXL (cs2762), anti- myeloid cell leukemia factor 1 (MCL1) (cs5453) and anti-glyceraldehyde-3-phospahte dehydrogenase (GAPDH) (cs2118), from Cell Signaling Technology, Danvers, MA, USA, anti-BCL2 interacting protein 3 (BNIP3) from Abcam, Cambridge, UK (Ab109362) and anti-microtubule associated protein 1 light chain 3 beta (LC3B) from Sigma (#7543). Chemiluminescence was used for visualization using the ECL Western blotting detection reagents (PerkinElmer, Waltham, MA, USA) according to the manufacturer. Densitometric analysis was performed using ChemiDoc MP Imaging system and ImageLab software version 5.0 (Bio-Rad Laboratories, Herkules, CA, USA).

### 2.14. RNA-Isolation and qRT-PCR

Total RNA from cell lines, adult mouse CM or iPSC-derived CM was prepared using Trizol (Life Technologies, Carlsbad, CA, USA), cDNA synthesis was performed with 1 μg of total RNA digested with DNaseI and subjected to SYBR green qRT-PCR (primer sequences are provided in Table 1) and TaqMan-based (Applied Biosystems, Waltham, MA, USA) gene expression profiling following the manufacturer’s protocol and as described [9,18]. Primer/probe assays for human BCLXL (Hs00236329_m1), human BNIP3 (Hs00969291_m1) and human 18S (Hs99999901_s1) were purchased from Applied Biosystems, with 18S serving as an internal control. Real-time PCR was performed using an ABI7500 cycler and the StepOne Software v2.3 (Applied Biosystems, Waltham, MA, USA).

### 2.15. Statistics

All the data are presented as mean ± SD unless otherwise stated. *p*-values were calculated using two-sided Student’s *t*-tests or 1-way ANOVA with Bonferroni’s post hoc correction for multiple group comparisons. For the statistical analysis of Kaplan–Meier survival curves, Log-rank tests were performed. *p* < 0.05 was considered significant. Data analysis was performed using GraphPad Prism version 7.0 for Mac OS X (GraphPad Software, San Diego, CA, USA).

## 3. Results

### 3.1. Acute Leukemia-Bearing Mice Developed Profound Cardiac Atrophy Associated with Impaired Cardiac Function

**Figure 1:**
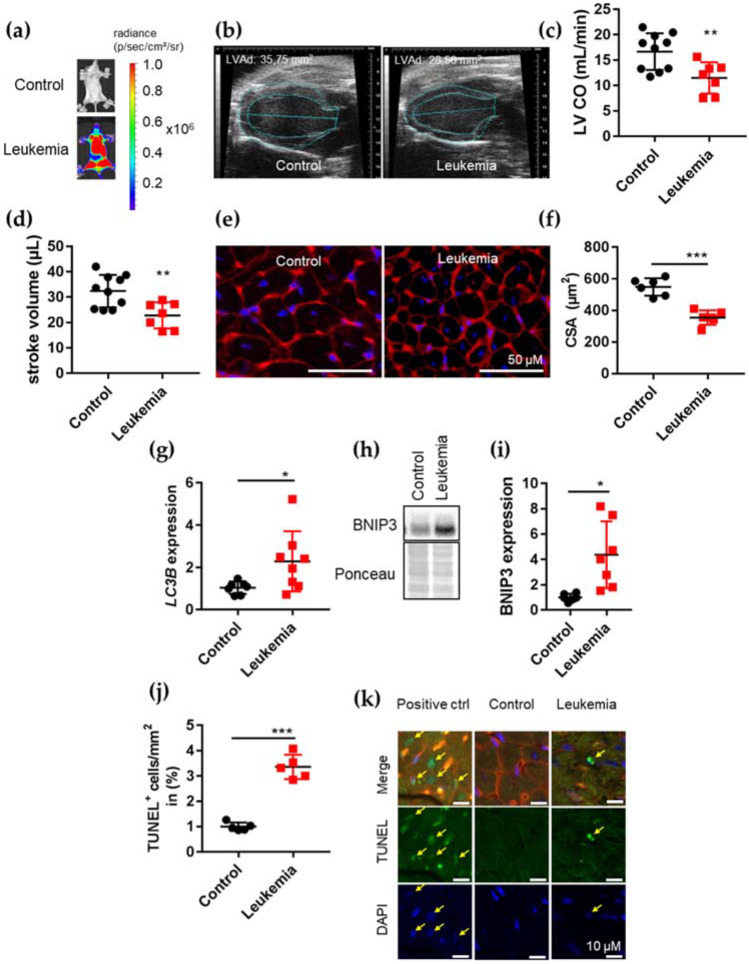
Cardiac phenotype of BCR-ABL+ ALL. (**a**) Representative BLI of NSG mice non-transplanted (control) or transplanted with luciferase-expressing BV173 cells four weeks after inoculation. (**b**) Representative echocardiographic picture in parasternal long axis view at end-diastole of BV173 and control heart at severe state of disease four weeks after transplantation. Left ventricular area diastole (LVAd) indicates cardiac dimensions and size. (**c**,**d**) Left ventricular cardiac output (LV CO) (**c**) and endocardial stroke volume (**d**) determined by echocardiography of age-matched healthy controls (control) or BV173-mice at severe state of disease four weeks after transplantation. ** *p* < 0.01. (**e**) LV cryosections stained with wheat germ agglutinin (WGA, cell membranes, red) and nuclei (DAPI, blue), scale bar: 50 µm. (**f**) Dot plot summarizes cardiomyocyte cross-sectional area (CSA) in BV173 (*n* = 6) and control (*n* = 5) LVs at severe state of disease four weeks after transplantation. *** *p* < 0.001. (**g**) qRT-PCR analysis of LC3B mRNA expression of control or BV173-mice LV at severe state of disease four weeks after transplantation * *p* < 0.05. (**h**,**i**) Representative immunoblot and densitometric quantification (*n* = 7) of LV tissue for BNIP3. Ponceau served as loading control. * *p* < 0.05. (**j**) Dot plot summarizes the number of TUNEL-positive nuclei per mm^2^ in BV173 (*n* = 5) and control (*n* = 5) LVs at severe state of disease four weeks after transplantation. *** *p* < 0.001. (**k**) Representative TUNEL positive nuclei (green) with apoptotic morphology co-stained with WGA (red) and DAPI (blue) (upper panel: merged staining TUNEL, WGA and DAPI; middle panel: TUNEL alone; lower panel: DAPI alone) of healthy control and BV173 NSG mice. Yellow arrows indicate double-positive stained cells for TUNEL and DAPI. Scale bars indicate 10 μm.

In the present study, we used the previously described BCR-ABL+ ALL cell line BV173 and patient-derived ALL xenografts (PDX) with stable luciferase expression, which allows non-invasive BLI to be used to visualize the leukemic burden in NSG mice (Figure 1a and Figure S1a) [9]. As described before, female NSG mice systemically engrafted with BV173 cells developed aggressive leukemia with a mean survival of 32 ± 2 days [9,10] (Figure 1a). At an advanced stage of leukemia (around week 4 after cell injection), mice displayed severe cardiac dysfunction, as assessed by echocardiography, in comparison with age-matched healthy controls (Figure 1b, Table 2).

This cardiac phenotype included a significantly reduced heart size and substantially impaired left ventricular (LV) function due to reduced cardiac output (LV CO) and stroke volume (ESV) (Figure 1b–d, Table 2). Post-mortem analysis revealed reduced heart weight (Table 2). Together with the reduced total body weight (Table 2), this phenotype suggests cachexia and cardiac atrophy in mice with advanced leukemia.

Accordingly, we observed a reduction in cardiomyocyte (CM) cross-sectional area (CSA) (Figure 1e,f) associated with enhanced cardiac expression of *LC3B* (Figure 1g and File S1) and BNIP3 both linked to cardiac atrophy in leukemic as compared with control mice (Figure 1h,i, File S1) [18,27,28]. Beyond cardiac atrophy, TUNEL assays revealed an increase in rare cardiomyocyte apoptosis in leukemic compared with control mice (Figure 1j,k). In line with the BV173 results, PDX engrafted female NSG mice developed aggressive leukemia associated with reduced heart size and weight, reduced cardiomyocyte CSA, impaired LV function, increased amount of TUNEL+ cardiac cells and cardiac BNIP3 expression (Figure S1b–h, File S1, Table 2).

### 3.2. Selective Ablation of Leukemia with Herpes Simplex Virus-Thymidine Kinase (HSV-TK)/Ganciclovir (GCV) Did Not Recover Cardiac Function and Output

**Figure 2:**
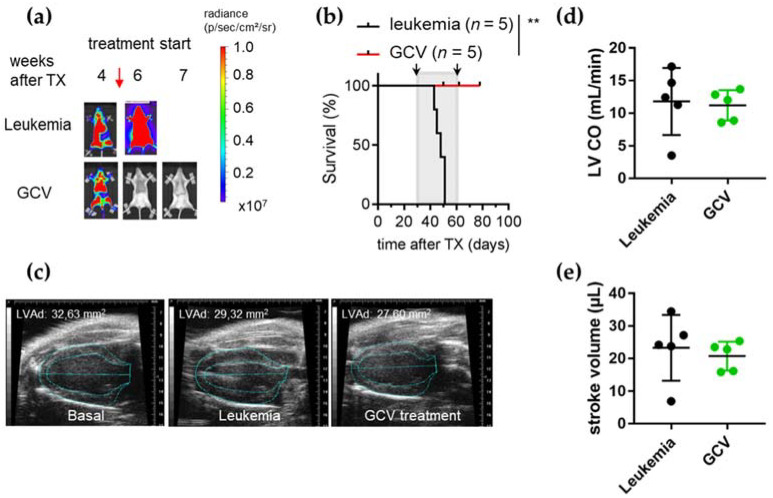
Severe cardiac impairment at late stages of ALL progression could not be reverted by the eradication of leukemia using GCV treatment. (**a**) Serial BLI of NSG mice transplanted with luciferase and HSV-TK expressing BV173 cells. Mice were monitored up to seven weeks after inoculation. GCV treatment started four weeks after transplantation. (**b**) Kaplan–Meier survival curve for untreated (leukemia *n* = 5) and GCV treated (*n* = 5) BV173-mice. Grey area indicates the GCV treatment period. Black arrows indicate time points of echocardiographic analysis. Log-rank test was used for statistical survival analysis. ** *p* < 0.01. (**c**) Representative serial echocardiographic pictures in parasternal long axis view at end-diastole of NSG hearts at basal state, severe leukemia disease state and after 28 days of GCV treatment. Heart size shown as LVAd. (**d**,**e**) Left ventricular cardiac output (LV CO) (**d**) and stroke volume (**e**) of BV173-mice determined by echocardiography at severe state of disease or after 28 days of GCV treatment.

We next used a genetic model to analyze the leukemia dependency of this cardiac phenotype. NSG mice were injected with BV173 cells stably expressing both luciferase and HSV-TK for chemotherapy-free selective ablation of transduced leukemic cells upon treatment with GCV [25]. Upon advanced leukemic engraftment at four weeks, mice were intraperitoneally treated with GCV, leading to the rapid depletion of leukemia and prolonged survival (Figure 2a,b).

However, no significant improvement in ALL-induced cardiac failure was detected three weeks after remission induction with no recovery of cardiac dimensions (decline in diastolic LV area (LVAd)) and cardiac dysfunction (impaired LV CO and ESV) at the end of GCV therapy (Figure 2c–e; Table 3).

The cardiotoxicity of GCV therapy could be excluded by the treatment of healthy mice with GCV for four weeks with no detectable effects on cardiac function and output (Table S1). These data demonstrate severe cardiac impairment at late stages of ALL progression, which is not reverted by the eradication of leukemia.

### 3.3. Combination Therapy of DAS/VEN/DEX Eradicated Leukemia and Improved Cardiac Function and Output

As we have shown previously, the chemotherapy-free combination therapy consisting of DAS/VEN/DEX induces long-term leukemia-free survival after initial treatment blocks of several weeks [9].

**Figure 3:**
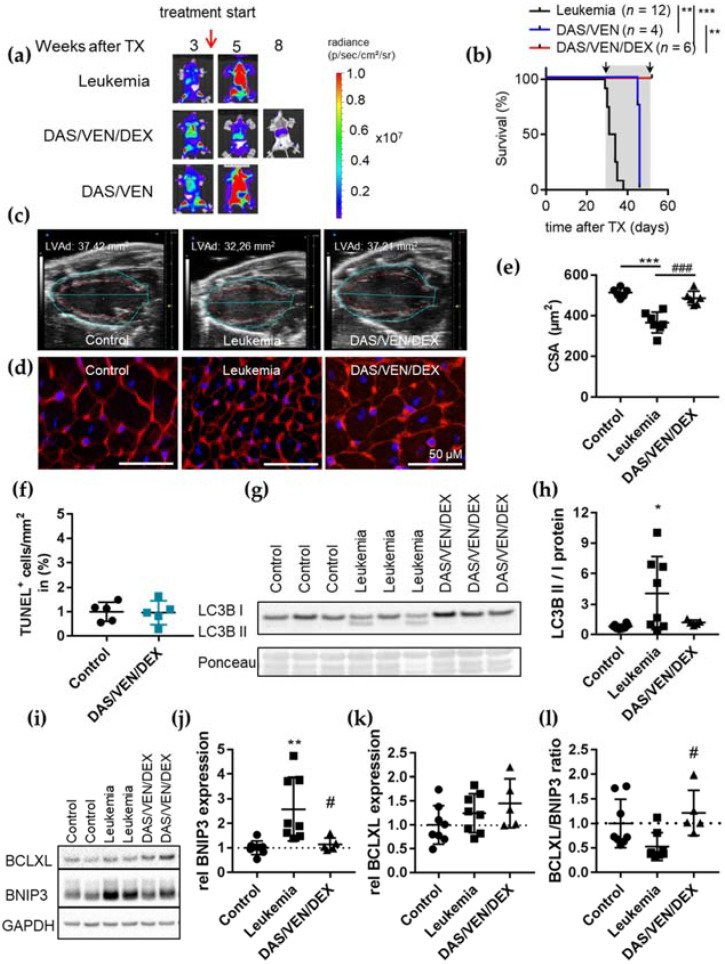
Treatment at advanced state of disease can restore cardiac function. (**a**) Representative serial BLI analysis of NSG mice transplanted with luciferase expressing BV173 cells. Mice were treated at advanced state of leukemia four weeks after transplantation with DAS/VEN/DEX (*n* = 6) or DAS/VEN (*n* = 4). (**b**) Kaplan–Meier survival curve for BV173-mice treated with DAS/VEN/DEX (*n* = 6) or DAS/VEN (*n* = 4). Grey area indicates the treatment period. Black arrows indicate timepoints of echocardiographic analysis. Log-rank test was used for statistical survival analysis. ** *p* < 0.01, *** *p* < 0.001. (**c**) Representative echocardiographic picture in parasternal long axis view at end-diastole of age-matched healthy control, untreated BV173 and DAS/VEN/DEX-treated BV173 hearts at four weeks after treatment start. LVAd indicate cardiac dimensions and size. (**d**) Representative LV cryosections stained with WGA (cell membranes, red) and nuclei (DAPI, blue), scale bar: 50 µm. (**e**) Dot plot summarizes cardiomyocyte CSA of healthy control (*n* = 6), untreated BV173 (*n* = 7) and DAS/VEN/DEX treated BV173 (*n* = 6) hearts at four weeks after treatment start. *** *p* < 0.001 vs. control, ^###^ *p* < 0.001 vs. leukemia. (**f**) Dot plot summarizing the number of TUNEL-positive nuclei per mm^2^ in healthy control (*n* = 5) and DAS/VEN/DEX treated BV173 (*n* = 5) hearts, not significant. (**g**,**h**) Immunoblot analysis and densitometric quantification of LV tissue of healthy (control) or BV173-mice treated with vehicle or DAS/VEN/DEX. Expression of LC3B isoforms was analyzed. Ponceau served as loading control. * *p* < 0.05. (**i**) Immunoblot analysis of LV tissue of healthy (control) or BV173-mice treated with vehicle or DAS/VEN/DEX. Expression of BCLXL and BNIP3 was analyzed. GAPDH served as loading control. (**j**–**l**) Densitometric quantification of BNIP3 (**j**), and BCLXL (**k**) and ratio of BCLXL/BNIP3 (**l**) expression. * *p* < 0.05 vs. control, ^#^ *p* < 0.05 vs. leukemia.

Therefore, we treated BV173-mice with advanced leukemia (at day 30 ± 1 post transplantation with BLI signal: total flux 1.2 × 10^12^ ± 0.7 × 10^12^ p/s) (Figure 3a) using either the DAS/VEN/DEX triple- or the DAS/VEN double-therapy for four weeks and analyzed cardiac function, output and dimension, as well as leukemic burden and survival. Therapy with DAS/VEN/DEX but not DAS/VEN led to leukemic regression and prolonged survival beyond the course of treatment (Figure 3b). In contrast to GCV treatment, therapy with DAS/VEN/DEX was associated with improved cardiac dimensions, heart weight and LV function analyzed in week four after the start of treatment (Figure 3c and Table S2). In addition, cardiomyocyte CSA was preserved (Figure 3d,e) and no induction of apoptosis was detected (Figure 3f), with no difference in cardiac phenotype compared to age-matched healthy controls. Mice treated with DAS/VEN double-therapy had a progressive leukemic burden and succumbed to leukemia under treatment.

In an experiment closer to the situation seen in the clinics for ALL patients, BV173-mice were treated with the DAS/VEN/DEX triple combination at an earlier leukemic stage (seven days post transplantation with BLI signal total flux 6.1 × 10^8^ ± 3.2 × 10^8^ p/s) and cardiac and morphometric analyses were performed in a follow-up cohort of long-term survivors after 38 weeks. No signs of cardiac dysfunction or alterations in cardiac and CM dimensions (Table S3) were detected, without any differences with age-matched healthy controls (Figure S2 a–e). These data clearly demonstrate that cardiac outcome depends on the kind of anti-leukemic therapy in this model.

### 3.4. DAS/VEN/DEX Alters the BCL2 Family Network Differentially in ALL Cells and Cardiomyocytes

The DAS/VEN/DEX triple combination has been optimized for apoptosis induction in ALL cells based on increased mitochondrial outer membrane permeabilization (MOMP) [9,10].

In the BCR-ABL+ ALL cell line, BV173, DEX and DAS enhance pro-apoptotic BIM expression and, at the same time, reduce anti-apoptotic BCLXL and MCL1 protein levels (Figure S3a). As a consequence, pro-apoptotic BIM loading to BCL2 is enhanced, which sensitizes these ALL cells to VEN cytotoxicity [9,10]. Hence, the combination of DAS/VEN/DEX is highly synergistic, even in primary ALL cells (Figure S3b).

Surprisingly, and in contrast to its effect on ALL cells, the DAS/VEN/DEX triple-therapy does not enhance the low level of apoptosis in the heart but rather induces cardiac recovery from ALL-induced damage (Figure 3c–f). BIM binding to BCL2 is the main predictor of VEN cytotoxicity in ALL cells. In CM, however, BIM is silenced during postnatal development, and adult CM does not rely on BCL2 for survival (Figure S3c) [29,30,31]. Therefore, we analyzed the cardiac expression of additional mitochondrial apoptosis regulators BCLXL and MCL1, as well as that of BNIP3 and LC3B involved in the regulation of cellular atrophy (Figure 3g–l and Figure S3d–f). BCLXL has a dual function as an anti-apoptotic protein and as a regulator for BNIP3 and LC3B [28,32,33,34]. BCLXL and MCL1 are expressed at similar levels in leukemic and control mice (Figure S3d,e). In contrast, we found heterogeneous but significantly increased ratios of LC3B isoforms II/I in LV tissue, indicating elevated numbers of autophagolysosome vesicles and increased cardiac BNIP3 levels in leukemic as compared to control mice (Figure 3g–j). In line with several reports that BNIP3 is epigenetically silenced in leukemia and diverse solid tumors [35,36,37], BNIP3 expression could not be detected in leukemic BV173 cells (Figure S3f). Upon DAS/VEN/DEX treatment, both enhanced cardiac BNIP3 expression and the increased ratio of LC3B isoforms II/I were reverted (Figure 3g–j). Furthermore, cardiac BCLXL expression increased upon DAS/VEN/DEX but not DAS/VEN treatment in vivo (Figure 3k,l and Figure S3g,h).

To further validate these findings, we analyzed the RNA expression of *BNIP3* and its binding partner *BCLXL* in cell cultures of primary adult mouse CM (Figure S3i,j) and human iPSC-derived CM (iPSC-CM) (Figure S3k,l) treated with DAS, VEN, and DEX alone or in combination. Only DEX, but neither VEN nor DAS, induced *BCLXL* expression in both adult and iPSC-CM. *BNIP3* expression was not affected by these drugs (Figure S3j and Figure 3l). These data demonstrate that therapies designed to optimize apoptosis induction in ALL may circumvent cardiac on-target side effects and even activate recovery from cellular atrophy in the heart.

## 4. Discussion

In the present study, we analyzed the impact of BCR-ABL+ ALL on the heart, as well as the effect of targeted anti-cancer therapy with DAS, DEX and VEN on the functional and molecular levels. To date, no cardiotoxic side effects have been reported for the treatment with VEN. In contrast, therapies with TKIs are associated with the development of cardiac complications and disorders [15,16].

The effects of dexamethasone on the cardiovascular system can be adverse as well as cardioprotective [38]. Multiple studies have reported the development of hypertrophy due to prolonged glucocorticoid treatment accompanied with atherosclerosis and even heart failure [39,40,41]. In contrast, especially in the setting of pathological events such as infarction or exposure to cardiotoxic drugs (e.g., doxorubicin), treatment with DEX has cytoprotective effects and inhibits the apoptosis of CM [42,43]. Most of these pro-survival effects of DEX have been associated with the induction of BCLXL expression [42,43]. Other factors that influence CM survival are an inhibition of TNFα signaling, reduction in metalloproteinases and reactive oxygen species or maintenance of sarcoplasmic reticulum Ca^2+^ homeostasis [44,45].

The combination of multiple anti-cancer drugs can lead to unknown adverse effects. Therefore, the safety of multiple combination therapies should at least be verified in animal models to avoid unexpected cardiac adverse events from anti-cancer therapies, even if such side effects may not detect human pathologies. Here, we used the BCR-ABL+ cell line BV173 and a PDX model in NSG mice that both developed aggressive leukemia associated with reduced heart weight and size, impaired LV function, reduced cardiomyocyte CSA and enhanced cardiac apoptosis. This phenotype of cardiac atrophy was associated with increased BNIP3 expression. BNIP3 is an atypical pro-apoptotic protein of the BCL2 protein family and plays a central role in apoptosis and autophagy of cardiomyocytes [32,33]. It regulates the apoptotic balance as an autophagy receptor by direct interaction with LC3 proteins on autophagosomes [34]. Accordingly, LC3B II/I ratios were also elevated in mice with severe leukemia.

We demonstrated that chemotherapy-free targeted therapies with DAS, DEX and VEN designed to optimize apoptosis induction in ALL cells [9,10] may circumvent cardiac on-target side effects and may even activate cardiac recovery. Moreover, we were able to show a genetic model with BV173 cells stably expressing both luciferase and HSV-TK for the chemotherapy-free selective ablation of transduced leukemic cells upon treatment with GCV, demonstrating that the cardiac impairment at late stages of ALL progression could not be reverted by the remission of leukemia [25]. Thus, the heart is not able to recover from leukemia-induced cardiac damage and harbors long-lasting cardiac side effects under these conditions. In contrast to our data, a recently published study has demonstrated that a reversible murine cancer model using B16F10 melanoma-HSV-TK cells enabling melanoma-cell-specific chemotherapy-free elimination by GCV treatment showed cardiac recovery [46]. In addition, after cardiac recovery, all melanoma-induced phenotypic, metabolic, molecular and functional changes in the heart were almost reversible after GCV-mediated tumor elimination without evidence of a contribution to long-term cardiac damage [46]. These data clearly demonstrate that, depending on the type of cancer, different strong and long-lasting cardiac complications and adverse effects may occur.

In contrast to GCV treatment, therapy with DAS/VEN/DEX efficiently eradicated leukemia and was associated with improved cardiac weights and dimensions and LV function. Moreover, no difference in cardiac phenotype has been found compared to age-matched healthy controls. However, double therapy with DAS/VEN failed to induce the remission of leukemia, and mice succumbed to progressive disease under treatment.

At the molecular level, low or absent expression of BIM and BCR-ABL in LV cardiac tissue minimizes the risk of on-target side effects in cardiomyocytes upon therapy with VEN and DAS, respectively. Furthermore, DEX acts differentially on the apoptotic potential of leukemic cells and cardiomyocytes. Although it induces pro-apoptotic BIM expression in leukemic blasts, and thereby contributes to efficient apoptosis induction in leukemic cells, it shows pro-survival potential in cardiomyocytes. On the one hand, BIM is silenced in cardiomyocytes during postnatal development and is, therefore, not induced by DEX treatment. On the other hand, anti-apoptotic BCLXL expression in the cardiac tissue of mice and in cardiomyocyte cell cultures is profoundly enhanced by DEX therapy and may thereby contribute to cardiac recovery from atrophy and protect from cardiomyocytic apoptosis. This is in line with several reports that, under pathological stress conditions such as infarction or treatment with cardiotoxic drugs (e.g., doxorubicin), the induction of BCLXL in CM by DEX has cytoprotective effects [42,43]. Furthermore, we did not see signs of CM hypertrophy—a reported side effect of DEX treatment—in our experiments in DAS/VEN/DEX treated mice after a treatment duration of four weeks in comparison with healthy control animals.

In addition, treatment with DAS/VEN/DEX normalized BNIP3 expression and the LC3 B II/I ratio in cardiac tissue, indicating cardiac recovery.

The combination therapy of DAS, DEX and VEN in NSG mice was started at a stage of advanced leukemia. Since ALL patients in the clinics would be treated at a much earlier timepoint, we used a more clinically relevant BV173 model by starting anti-leukemic combination therapy at a much earlier timepoint (one week after cell injection). In this follow-up cohort of long-term survivors (after 38 weeks), no signs of cardiac dysfunction or alterations in cardiac and CM dimensions were found, without any differences from age-matched healthy controls. Therefore, and in contrast to GCV-treatment, cardiac outcome depends on the kind of anti-leukemic therapy in this model.

Although cell-, tissue-, organ- and species-specific effects, and their underlying molecular mechanisms, remain to be determined in detail, these data demonstrate that murine xenograft leukemia models can be used to simultaneously optimize anti-leukemic therapies and analyze disease- and/or therapy-dependent cardiotoxicity. The cardiac comorbidity of current anti-tumor therapies may have a tremendous impact on the long-term survival and morbidity of tumor patients, as was most prominently described for anthracycline-induced cardiomyopathy [47]. Transient cardiac damage during anti-leukemic therapies, as indicated by elevation of cardiac stress or ischemia markers such as N-terminal pro-brain natriuretic peptide (NT-proBNP) and troponin T, as well as arrhythmias, are regularly seen in patients during intensive anti-leukemic therapies [48]. Therefore, it seems reasonable to complement the careful clinical monitoring of cardiotoxicity in leukemic patients with further characterization of organ-specific side effects and signaling pathways activated by malignancy and/or anti-tumor therapies in the future.

## 5. Conclusions

In conclusion, our data provide evidence that BCR-ABL+ ALL induces severe and persisting cardiac damage with cardiomyocyte atrophy and functional impairment. The DAS/VEN/DEX triple-therapy not only eradicated the BCR-ABL+ ALL by the highly efficient induction of apoptosis in leukemic cells but also prevented cardiac damage and improved cardiac recovery. Leukemic animal models could be useful tools to optimize anti-leukemic therapy and monitor the safety of combination therapies to avoid unexpected adverse cardiac events from anti-cancer therapies.

## Figures and Tables

**Table 1:** SYBR Green qRT-PCR Primer sequences.

mRNA	Sense Primers (5′ to 3′)	Antisense Primers (5′ to 3′)
mmu 18S	GTAACCCGTTGAACCCCATT	CCATCCAATCGGTAGTAGCG
mmu BCLXL	GACAAGGAGATGCAGGTATTGG	TCCCGTAGAGATCCACAAAAGT
mmu BNIP3	CAGCATGAGAAACACAAGCGT	GTTGTCAGACGCCTTCCAATG
mmu LC3B	CATGCCGTCCGAGAAGACCT	TCGCTCTATAATCACTGGGATCT

**Table 2:** Morphometry and cardiac function of NSG mice transplanted with BCR-ABL+ ALL.

Parameters	Age-Matched Healthy Control for BV173 (*n* = 10)	BV173 (*n* = 7)	Age-Matched Healthy Control for PDX (*n* = 9)	PDX (*n* = 5)
BW (g)	24.12 ± 1.68	18.53 ± 1.85 *	28.06 ± 1.01	22.10 ± 1.81 ***
HW (mg)	98.46 ± 0.88	68.46 ± 8.29 ***	102.46 ± 6.83	86.20 ± 5.07 **
HW/BW (mg/g)	4.29 ± 0.55	3.7 ± 0.47	3.65 ± 0.15	3.92 ± 0.39
LVEDD (mm)	7.71 ± 0.64	6.65 ± 0.36 **	7.56 ± 0.37	7.19 ± 0.78
LVESD (mm)	6.35 ± 0.51	5.33 ± 0.68	6.48 ± 0.45	6.41 ± 0.31
EDA (mm^2^)	0.20 ± 0.02	0.16 ± 0.015 **	0.20 ± 0.01	0.16 ± 0.03 *
ESA (mm^2^)	0.09 ± 0.02	0.07 ± 0.02	0.11 ± 0.02	0.10 ± 0.02
HR (bpm)	511 ± 30	499 ± 34	506 ± 37	492 ± 36
EVd (µL)	43.87 ± 7.75	31.90 ± 5.58 *	43.44 ± 5.39	32.39 ± 9.84 *
EVs (µL)	11.36 ± 3.24	9.09 ± 4.33	15.75 ± 3.80	12.58 ± 4.60
FAC (%)	54.36 ± 5.56	53.28 ± 11.56	44.80 ± 7.80	41.60 ± 7.96
EF (%)	74.14 ± 5.58	71.84 ± 11.96	63.66 ± 7.80	61.51 ± 9.72

Body weight (BW), heart weight (HW), left ventricular end-diastolic diameter (LVEDD), left ventricular end-systolic diameter (LVESD), end-diastolic area (EDA), end-systolic area (ESA), heart rate (HR), endocardial diastolic volume (EVd), endocardial systolic volume (EVs), fractional area change (FAC), ejection fraction (EF) determined in NSG mice (cell lines BV173 and PDX). Age-matched healthy control mice for BV173-mice (short latency) and PDX-mice (long latency) were 15 and 28 weeks old, respectively. * *p* < 0.05, ** *p* < 0.01, *** *p* < 0.001 Student’s *t*-test to corresponding aged-matched healthy control group.

**Table 3:** Morphometry and cardiac function of NSG mice transplanted with BCR-ABL+ ALL. After confirmed state of advanced disease mice were treated with GCV for four weeks.

Parameters	Before Treatment		Four Weeks Treatment	
	Control (*n* = 14)	Leukemia (*n* = 5)	Control (*n* = 6)	GCV (*n* = 5)
LVEDD (mm)	7.62 ± 0.39	7.26 ± 0.48	7.98 ± 0.15	7.12 ± 0.62
LVESD (mm)	6.12 ± 0.89	6.44 ± 0.70	6.63 ± 0.48	5.97 ± 0.16 *
EDA (mm^2^)	0.19 ± 0.02	0.16 ± 0.05 *	0.20 ± 0.03	0.15 ± 0.02 *
ESA (mm^2^)	0.09±0.02	0.08 ± 0.02	0.09 ± 0.02	0.07 ± 0.01 *
HR (bpm)	505 ± 38	507 ± 28	500 ± 32	541 ± 13 *
LV CO (mL/min)	14.84 ± 2.44	11.8 ± 5.15	16.34 ± 4.29	11.19 ± 2.33 *
ESV (µL)	29.49 ± 4.87	23.3 ± 10.11	32.47 ± 7.32	20.72 ± 4.42 *
EVd (µL)	41.40 ± 7.91	31.73 ± 13.5	43.82 ± 8.53	27.53 ± 5.82 **
EVs (µL)	11.91 ± 5.15	8.43 ± 3.99	11.35 ± 3.74	6.81 ± 1.82 *
FAC (%)	52.37 ± 10.10	51.22 ± 6.44	54.42 ± 6.88	54.82 ± 3.16
EF (%)	72.03 ± 9.31	72.35 ± 7.5	74.04 ± 7.05	75.31 ± 3.64

After confirmed state of advanced disease mice were treated with GCV for four weeks. Left ventricular end-diastolic diameter (LVEDD), left ventricular end-systolic diameter (LVESD), end-diastolic area (EDA), end-systolic area (ESA), heart rate (HR), left ventricular cardiac output (LV CO), endocardial stroke volume (EVS), endocardial diastolic volume (EVd), endocardial systolic volume (EVs), fractional area change (FAC), ejection fraction (EF); * *p* < 0.05, ** *p* < 0.01 Student’s *t*-test to corresponding aged matched healthy control group.

## Data Availability

The data presented in this study are available in the Supplementary Materials File.

## Supplementary Materials

The following are available online at https://www.mdpi.com/article/10.3390/cancers14040983/s1, Figure S1: Representative BLI of NSG mice non-transplanted or transplanted with luciferase expressing PDX (L4951) cells 10 weeks after transplantation, Figure S2: Representative echocardiographic picture in parasternal long axis view at end-diastole of healthy control and DAS/VEN/DEX-treated BV173 hearts at 35 weeks follow-up (FU) after transplantation, Figure S3: Representative Immunoblot of BV173 cells treated for 24 h with or without DAS/VEN/DEX (each 20nM). GAPDH served as loading control, Table S1: Toxicity study four weeks of treatment in non-transplanted NSG mice, Table S2: Morphometry and cardiac function of NSG mice transplanted with BCR-ABL+ ALL, Table S3: Morphometry and cardiac function of long-term survivors, File S1: The Uncropped Western Blot Images for all the cropped pictures presented in the main manuscript and the supplementary materials.
