# Supplementary material for: Chemotherapy-Free Targeted Anti-BCR-ABL+ Acute Lymphoblastic Leukemia Therapy May Benefit the Heart
Source: Cancers (Basel). 2022 Feb 15;14(4):983. doi: 10.3390/cancers14040983 (PMC8870618; doi:10.3390/cancers14040983)
Supplement: Supplementary file 1 [file cancers-14-00983-s001.zip › cancers-1533146 - supplementary.pdf]

Supplementary Material: Chemotherapy-Free Targeted Anti-BCR-ABL+ Acute Lymphoblastic Leukemia Therapy May Benefit the Heart

Hanna Kirchhoff, Melanie Ricke-Hoch, Katharina Wohlan, Stefan Pietzsch, Ümran Karsli, Sergej Erschow, Robert Zweigerdt, Arnold Ganser, Matthias Eder, Michaela Scherr and Denise Hilfiker-Kleiner

Table S1. Toxicity study 4 weeks of treatment in non-transplanted male NSG mice.

Parameters	Control (*n* = 7)	GCV (*n* = 6)
LVEDD [mm]	7.65 ± 0.36	8.06 ± 0.44
LVESD [mm]	6.70 ± 0.26	6.92 ± 0.34
EDA [mm²]	0.20 ± 0.03	0.22 ± 0.02
ESA [mm²]	0.10 ± 0.01	0.12 ± 0.01
HR [bpm]	504 ± 35	528 ± 34
LV CO [ml/min]	15.49 ± 4.09	19.39 ± 4.18
ESV [µL]	30.71 ± 7.69	36.57 ± 6.59
EVd [µL]	44.26 ± 9.38	53.29 ± 8.90
EVs [µL]	13.55 ± 3.32	16.71 ± 3.86
FAC [%]	48.18 ± 6.27	48.17 ± 5.16
EF [%]	69.10 ± 6.78	68.66 ± 4.93

Mice were treated with 75 mg/kg/d GCV 5 days/week for 4 weeks. Left ventricular end-diastolic diameter (LVEDD), left ventricular end-systolic diameter (LVESD), end-diastolic area (EDA), end-systolic area (ESA), heart rate (HR), left ventricular cardiac output (LV CO), endocardial stroke volume (EVS), endocardial diastolic volume (EVd), endocardial systolic volume (EVs), fractional area change (FAC), ejection fraction (EF); no significance Student's *t*-test.

Table S2. Morphometry and cardiac function of NSG mice transplanted with BCR-ABL+ ALL. After confirmed state of advanced disease mice were treated with DAS/VEN/DEX for 4 weeks.

Variable	Before Treatment		4 Weeks Treatment	
	Control (*n* = 14)	Leukemia (*n* = 5)	Control (*n* = 6)	DAS/VEN/DEX (*n* = 5)
BW [g]	-	-	24.62 ± 1.34	19.20 ± 3.15 ***
HW [mg]	-	-	102.50 ± 7.94	98.86 ± 7.23
HW/BW [mg/g]	-	-	4.17 ± 0.35	5.14 ± 0.32 **
LVEDD [mm]	7.62 ± 0.39	7.63 ± 0.15	7.98 ± 0.15	7.82 ± 0.31
LVESD [mm]	6.12 ± 0.89	6.32 ± 0.33	6.63 ± 0.48	6.43 ± 0.53
EDA [mm²]	0.19 ± 0.02	0.18 ± 0.02	0.20 ± 0.03	0.18 ± 0.02
ESA [mm²]	0.09 ± 0.02	0.09 ± 0.02	0.09 ± 0.02	0.08 ± 0.02
HR [bpm]	505 ± 38	541 ± 26	500 ± 32	551 ± 19 *
LV CO [mL/min]	14.84 ± 2.44	11.87 ± 2.52 *	16.34 ± 4.29	13.69 ± 2.28
ESV [µL]	29.49 ± 4.87	22.07 ± 5.23 *	32.47 ± 7.32	24.91 ± 4.38
EVd [µL]	41.40 ± 7.91	35.46 ± 9.1	43.82 ± 8.53	34.25 ± 6.69
EVs [µL]	11.91 ± 5.15	13.39 ± 5.07	11.35 ± 3.74	9.34 ± 2.95
FAC [%]	52.37 ± 10.10	47.56 ± 8.68	54.42 ± 6.88	56.36 ± 6.76
EF [%]	72.03 ± 9.31	62.97 ± 7.8	74.04 ± 7.05	73.03 ± 4.49

Body weight (BW), heart weight (HW), left ventricular end-diastolic diameter (LVEDD), left ventricular end-systolic diameter (LVESD), end-diastolic area (EDA), end-systolic area (ESA), heart rate (HR), left ventricular cardiac output (LV CO), endocardial stroke volume (EVS), endocardial diastolic volume (EVd), endocardial systolic volume (EVs), fractional area change (FAC), ejection fraction (EF); * $p < 0.05$, ** $p < 0.01$, *** $p < 0.001$ Student's *t*-test to corresponding aged matched healthy control group.

Table S3. Morphometry and cardiac function of long-term survivors.

Variable	Control (*n* = 5)	FU DAS/VEN/DEX (*n* = 4)
BW [g]	28.06 ± 1.01	25.38 ± 2.98
HW [mg]	102.46 ± 6.83	90.18 ± 3.56
HW/BW [mg/g]	3.65 ± 0.15	3.56 ± 0.08
LVEDD [mm]	7.34 ± 0.21	7.53 ± 0.75

LVESD [mm]	6.27 ± 0.42	6.49 ± 0.51
EDA [mm²]	0.19 ± 0.01	0.19 ± 0.03
ESA [mm²]	0.10 ± 0.01	0.10 ± 0.02
HR [bpm]	531 ± 20	513.75 ± 8.66
EVd [µL]	43.08 ± 5.20	40.67 ± 9.91
EVs [µL]	14.55 ± 1.86	13.23 ± 3.93
FAC [%]	46.88 ± 6.89	47.12 ± 4.70
EF [%]	65.63 ± 7.19	67.51 ± 4.69

Follow-up (FU) of NSG mice transplanted with BV173 and treated six weeks with DAS/VEN/DEX 38 weeks after TX. Body weight (BW), heart weight (HW), left ventricular end-diastolic diameter (LVEDD), left ventricular end-systolic diameter (LVESD), end-diastolic area (EDA), end-systolic area (ESA), heart rate (HR), endocardial diastolic volume (EVd), endocardial systolic volume (EVs), fractional area change (FAC), ejection fraction (EF)no significance Student's *t*-test.

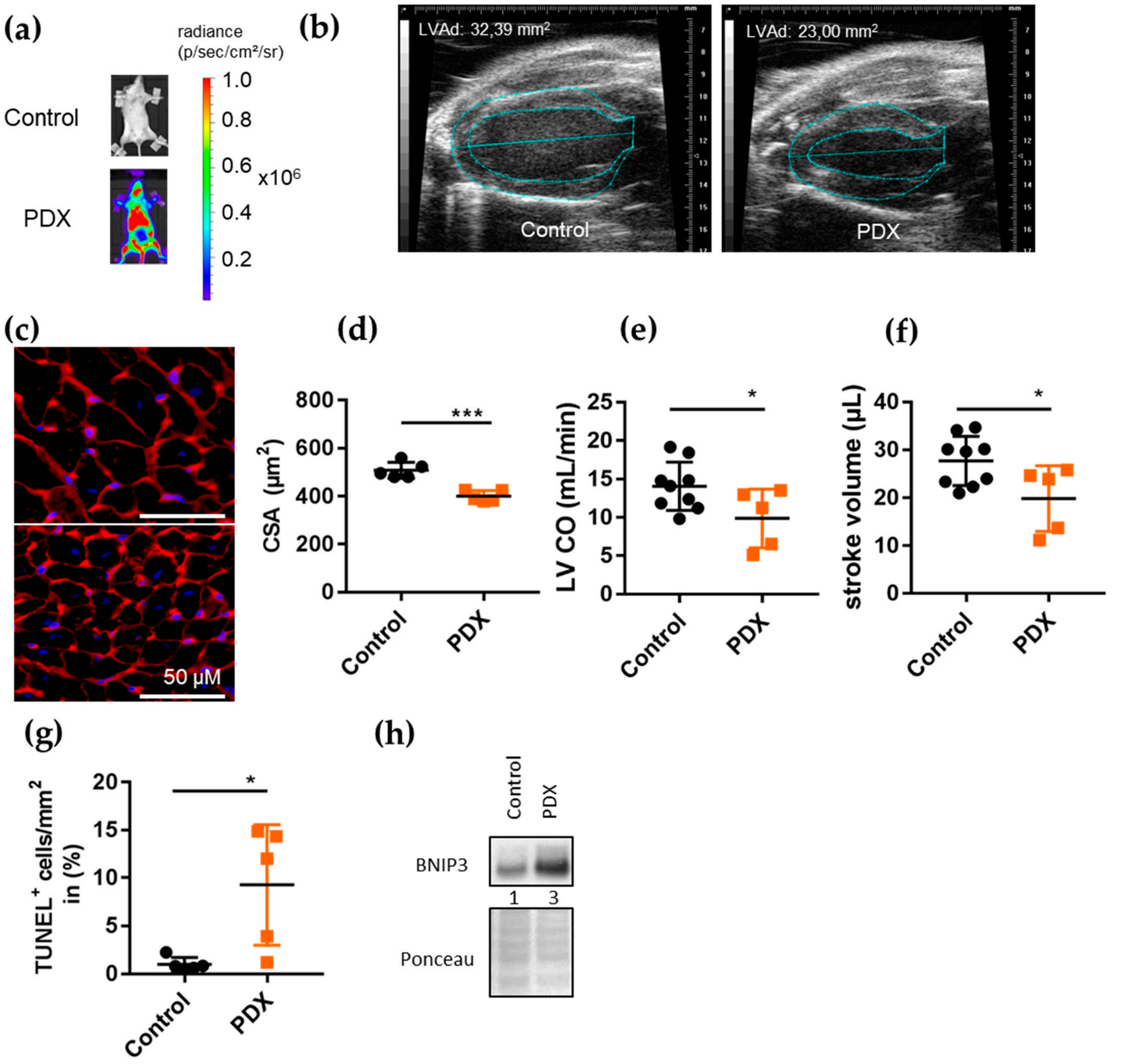

Figure S1: (**a**) Representative BLI of NSG mice non-transplanted or transplanted with luciferase expressing PDX (L4951) cells 10 weeks after transplantation. (**b**) Representative echocardiographic picture in parasternal long axis view at end-diastole of PDX (L4951) and control hearts at severe state of disease 10 weeks after transplantation. LVAd indicates cardiac dimension and size. (**c**)

Representative LV cryosections stained with WGA (cell membranes, red) and nuclei (DAPI, blue), scale bar: 50 µm. (**d**) Dot plot summarizes cardiomyocyte CSA in PDX (n = 5) and control (n = 5) LVs at severe state of disease 10 weeks after transplantation. *** $p < 0.001$. (**e**–**f**) Left ventricular cardiac output (LV CO) (**d**) and stroke volume (**e**) of age matched healthy controls (control) (n = 9) or PDX-mice (n = 5) 10 weeks after transplantation. (**g**) Dot plot summarize the number of TUNEL positive nuclei per mm^2 in PDX (n = 5) and healthy control (n = 5), * $p < 0.05$. (**h**) Representative Immunoblot of LV tissue of age matched healthy control mice or PDX-mice at severe state of disease for BNIP3. Ponceau served as loading control.

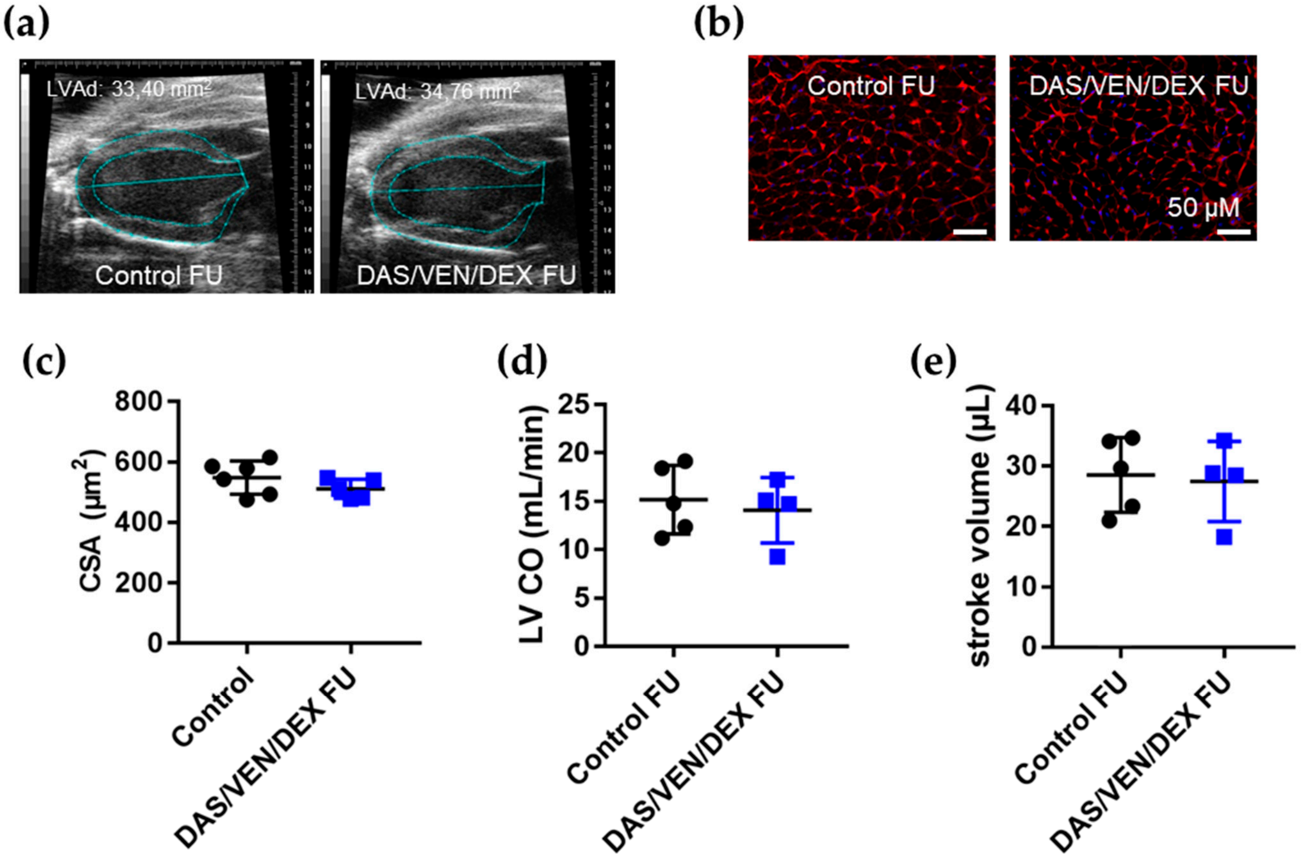

Figure S2. (**a**) Representative echocardiographic picture in parasternal long axis view at end-diastole of healthy control and DAS/VEN/DEX treated BV173 hearts at 35 weeks follow-up (FU) after transplantation. LVAd indicate cardiac dimensions and size. (**b**) Representative LV cryosections stained with WGA (cell membranes, red) and nuclei (DAPI, blue), scale bar: 50 µm. (**c**) Dot plot summarizes cardiomyocyte CSA of healthy control (n = 6), untreated BV173 (n = 5) and DAS/VEN/DEX treated BV173 (n = 5) hearts at 35 weeks FU after transplantation. (**d**–**e**) Left ventricular cardiac output (LV CO) (**d**) and stroke volume (**e**) of age matched healthy controls (control) (n = 5) or follow-up mice treated with DAS/VEN/DEX (n = 4) 35 weeks after transplantation.

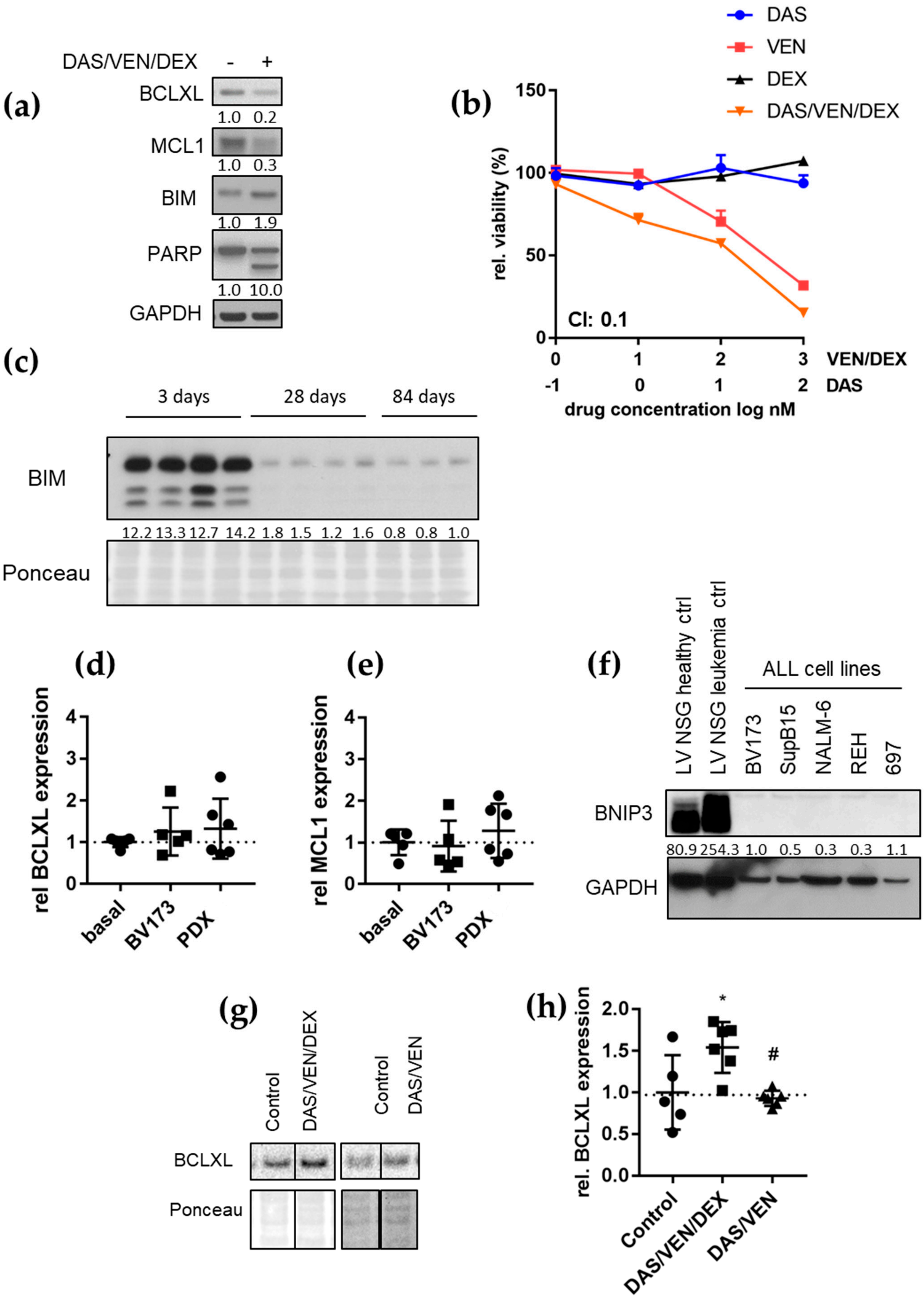
(a)
DAS/VEN/DEX - +
BCLXL
1.0 0.2
MCL1
1.0 0.3
BIM
1.0 1.9
PARP
1.0 10.0
GAPDH
(b)
DAS
VEN
DEX
DAS/VEN/DEX
rel. viability (%)
CI: 0.1
VEN/DEX
DAS
drug concentration log nM
(c)
3 days
28 days
84 days
BIM
12.2 13.3 12.7 14.2 1.8 1.5 1.2 1.6 0.8 0.8 1.0
Ponceau
(d)
rel BCLXL expression
basal
BV173
PDX
(e)
rel MCL1 expression
basal
BV173
PDX
(f)
LV NSG healthy ctrl
LV NSG leukemia ctrl
ALL cell lines
BV173
SupB15
NALM-6
REH
697
BNIP3
80.9 254.3 1.0 0.5 0.3 0.3 1.1
GAPDH
(g)
Control
DAS/VEN/DEX
Control
DAS/VEN
BCLXL
Ponceau
(h)
rel. BCLXL expression
*
#
Control
DAS/VEN/DEX
DAS/VEN

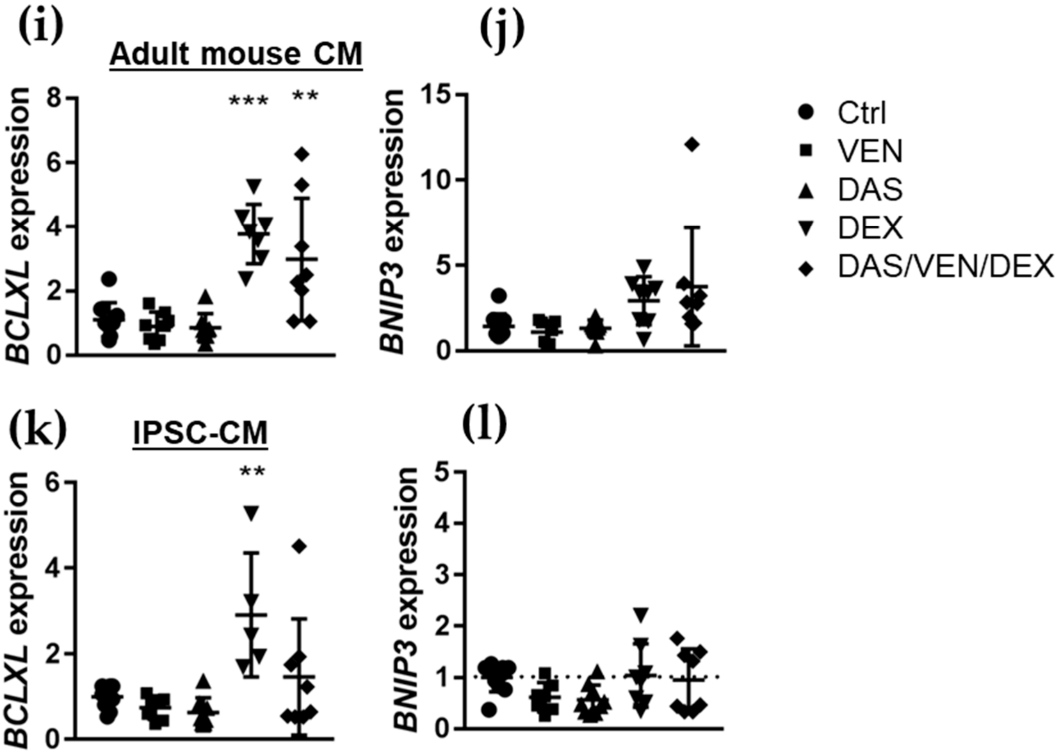

Figure S3: (**a**) Representative Immunoblot of BV173 cells treated for 24 h with or without DAS/VEN/DEX (each 20 nM). GAPDH served as loading control. (**b**) Cytotoxic effects of DAS, VEN and DEX on primary BCR-ABL+ ALL cells. ALL cells were plated 4 h prior to drug treatment onto primary MSCs. Cocultures were treated with increasing concentrations of DAS, VEN and DEX as indicated alone or in combination in fixed ratios. Cytotoxicity was determined by flow cytometric analysis of CD19 antibody and propidium iodide staining. Combination index (CI) at IC50 was calculated using CompuSyn software. CI < 1 was considered synergistic. (**c**) BIM Immunoblot of LV tissue of 3 days, 4 weeks and 3-month-old mice. Ponceau served as loading control. (**d**–**e**) Densitometric analysis of BCLXL (**d**) and MCL1 (**e**) expression in LV tissue of healthy NSG mice (n = 5) or BV173- (n = 5) and PDX-mice (n = 6). Quantification was normalized to Ponceau loading control. (**f**) BNIP3 Immunoblot of LV tissue of NSG mice and ALL cell lines (BV173, SUPB15, NALM-6, REH, 697). GAPDH served as loading control. (**g**–**h**) Immunoblot and densitometric quantification of LV tissue of NSG mice treated with vehicle (n = 5), DAS/VEN/DEX (n = 6) or DAS/VEN (n = 6) for 4 weeks. * $p < 0.05$ vs Control, # $p < 0.05$ vs Leukemia. (**i**–**j**) BCLXL and BNIP3 expression of adult mouse CM treated with DAS, VEN, DEX or a combination. *** $p < 0.001$, ** $p < 0.01$. (**k**–**l**) BCLXL and BNIP3 expression of iPS-CM treated with DAS, VEN, DEX or a combination. ** $p < 0.01$

File S1. The Uncropped Western Blot Images for all the cropped pictures presented in the main manuscript and the supplementary materials. Can be found as separate PDF file.

Publisher's Note: MDPI stays neutral with regard to jurisdictional claims in published maps and institutional affiliations.

© 2022 by the authors. Licensee MDPI, Basel, Switzerland. This article is an open access article distributed under the terms and conditions of the Creative Commons Attribution (CC BY) license (http://creativecommons.org/licenses/by/4.0/).
