# Supplementary material for: Chemotherapy-Free Targeted Anti-BCR-ABL+ Acute Lymphoblastic Leukemia Therapy May Benefit the Heart
Source: Cancers (Basel). 2022 Feb 15;14(4):983. doi: 10.3390/cancers14040983 (PMC8870618; doi:10.3390/cancers14040983)

Figure 1H

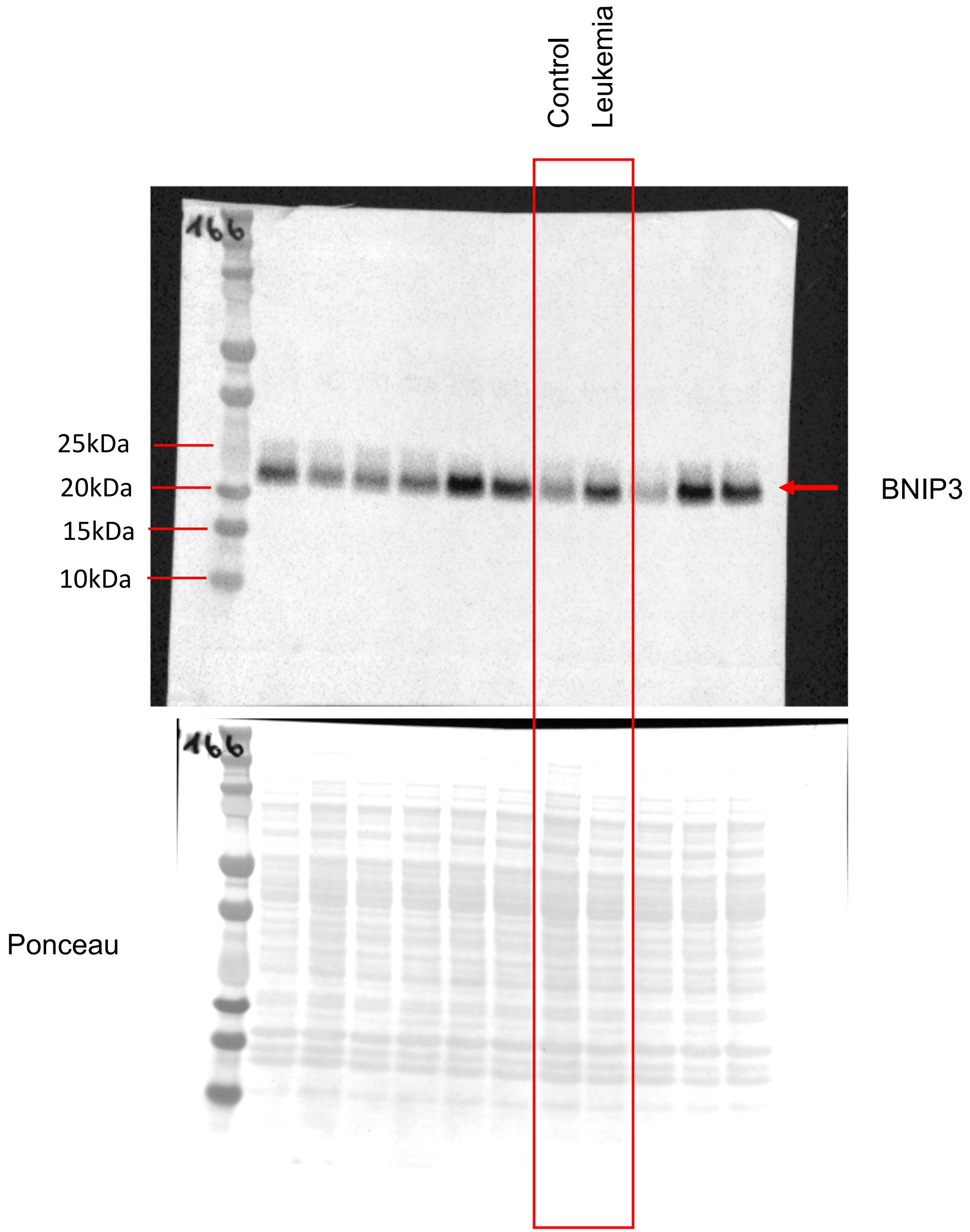

Figure 2G

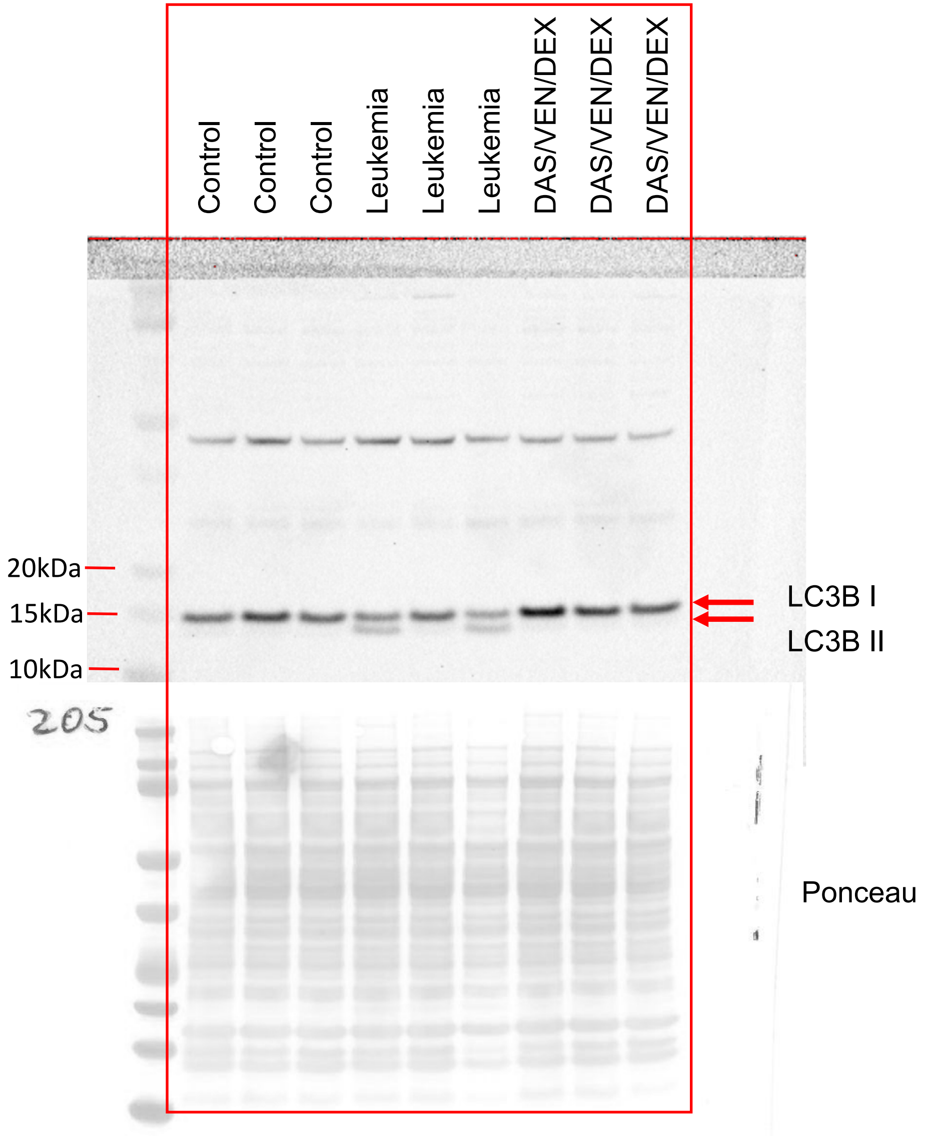
Control
Control
Control
Leukemia
Leukemia
Leukemia
DAS/VEN/DEX
DAS/VEN/DEX
DAS/VEN/DEX
20kDa
15kDa
10kDa
LC3B I
LC3B II
205
Ponceau

Figure 2I

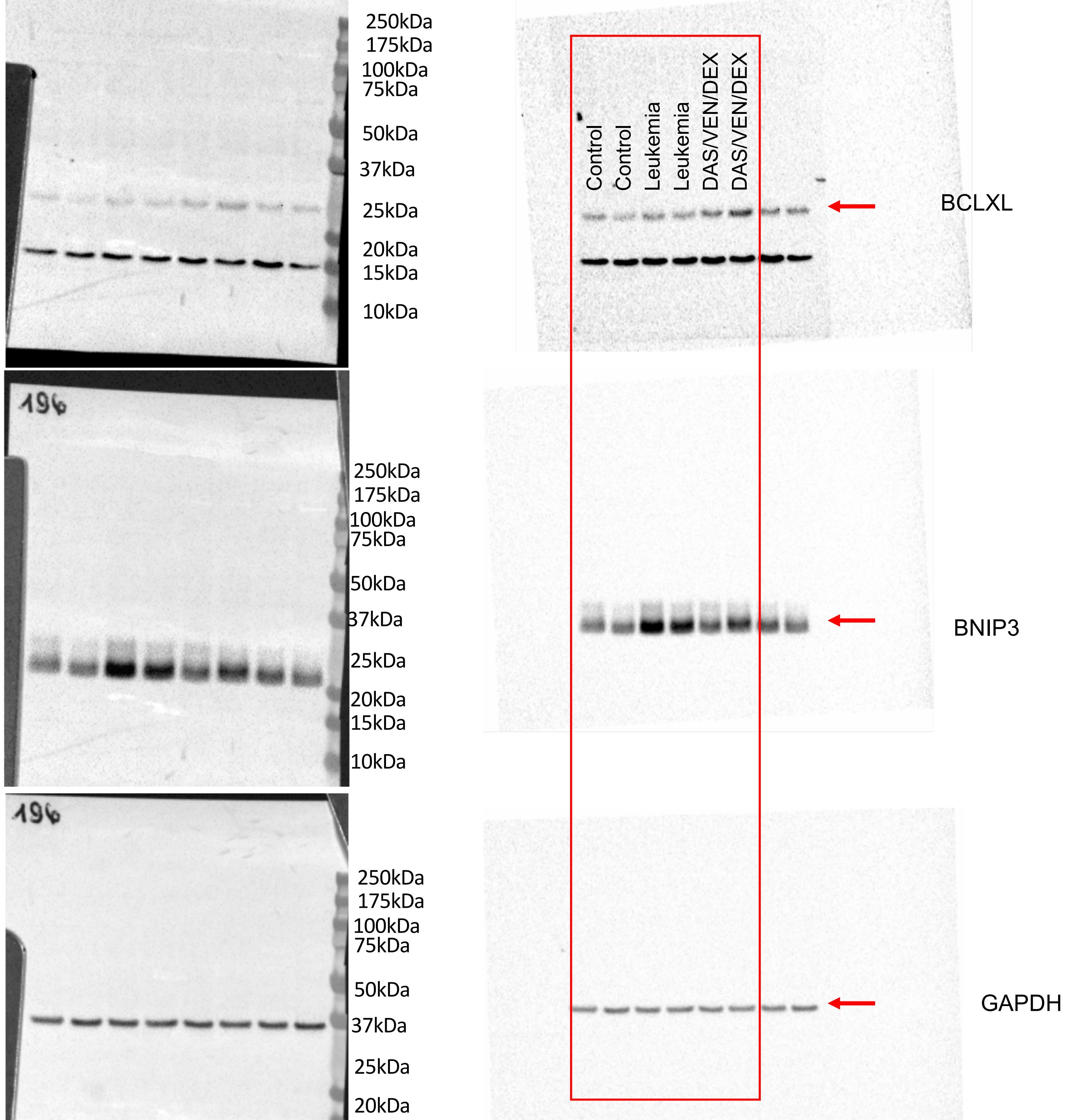
250kDa
175kDa
100kDa
75kDa
50kDa
37kDa
25kDa
20kDa
15kDa
10kDa
Control
Control
Leukemia
Leukemia
DAS/VEN/DEX
DAS/VEN/DEX
BCLXL
196
BNIP3
GAPDH

Supplemental Figure 1I

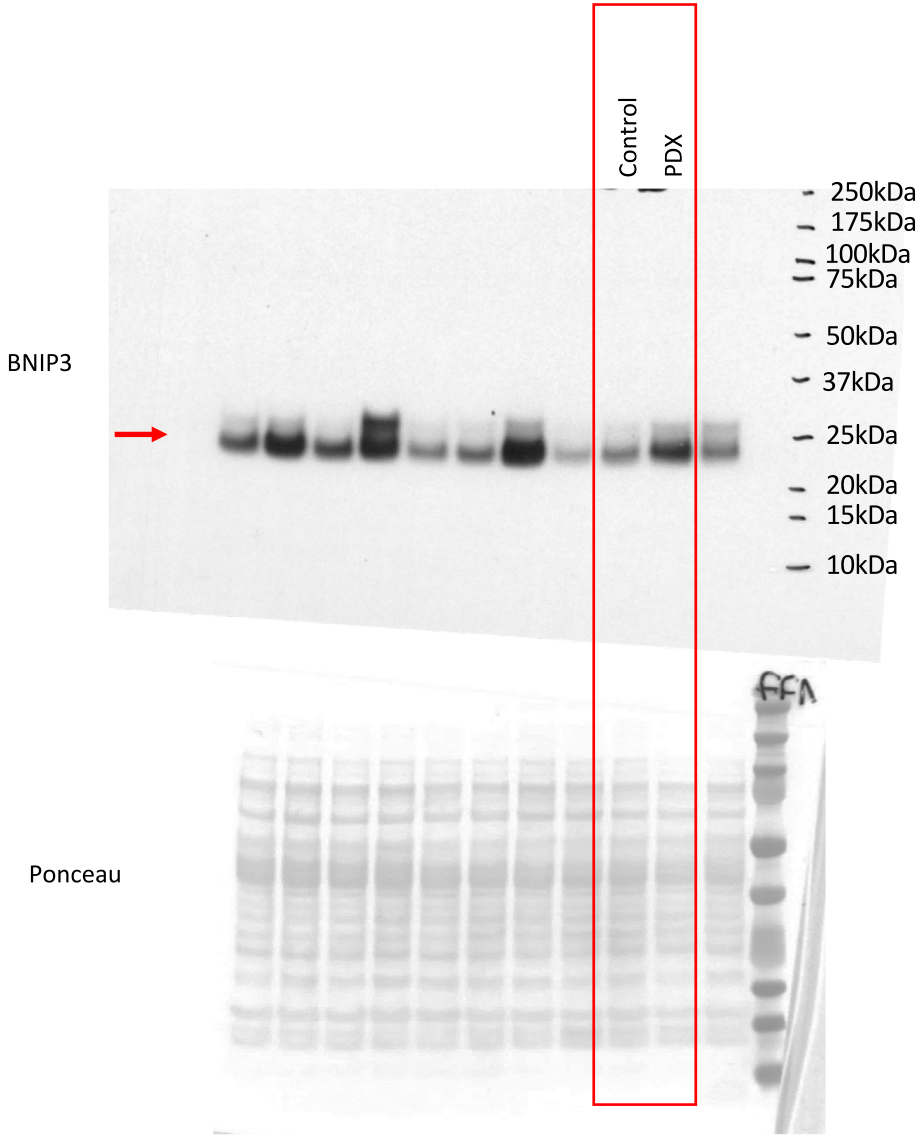

Supplemental Figure 3A

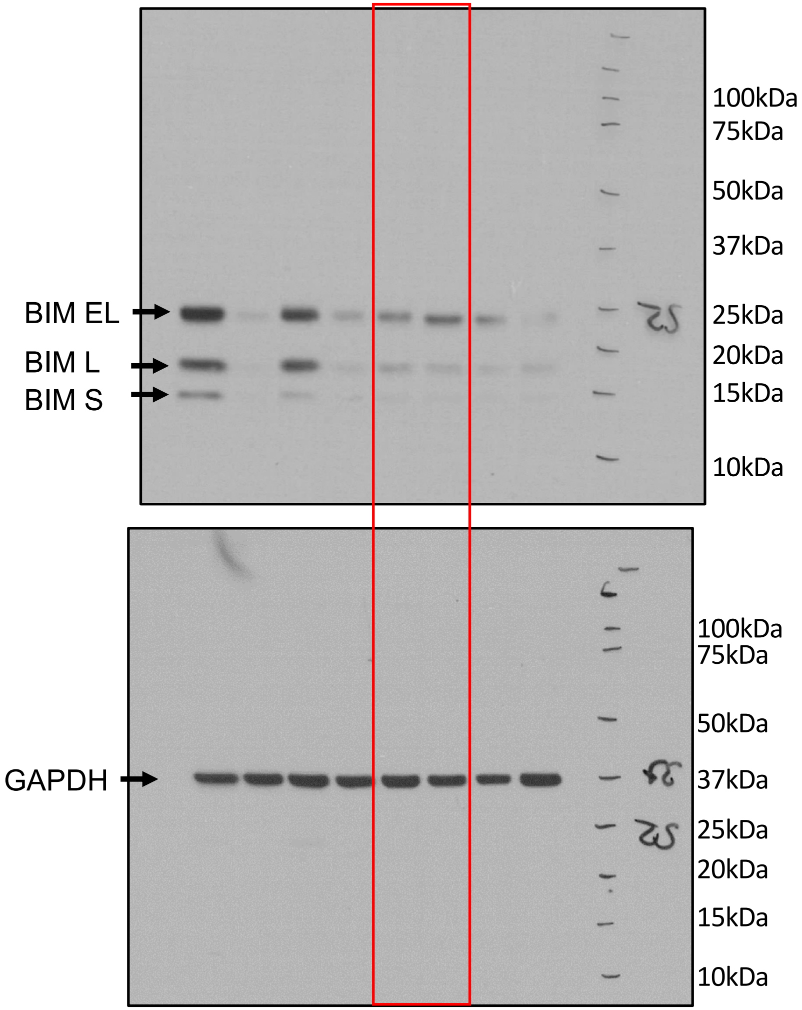

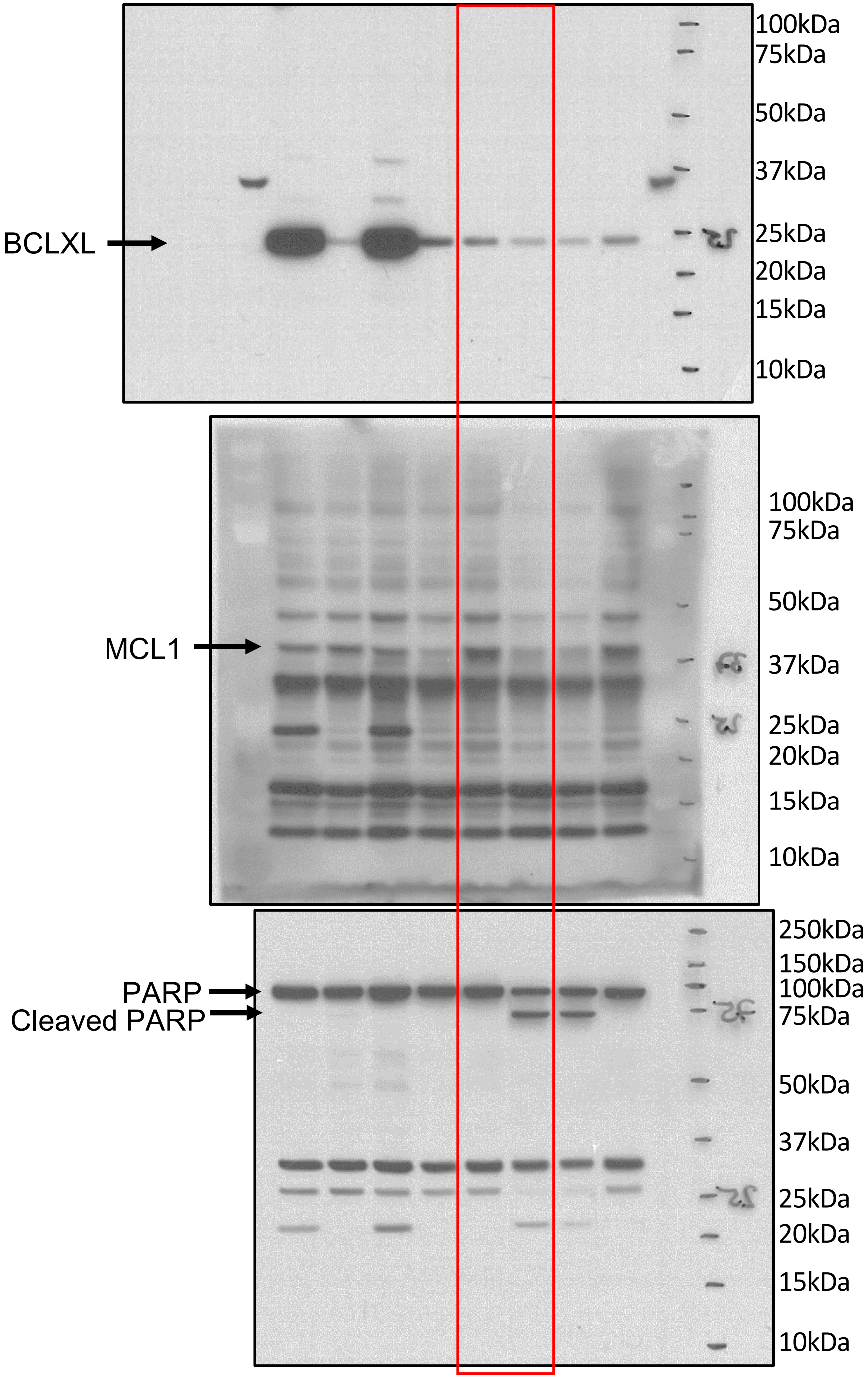

Supplemental Figure 3C

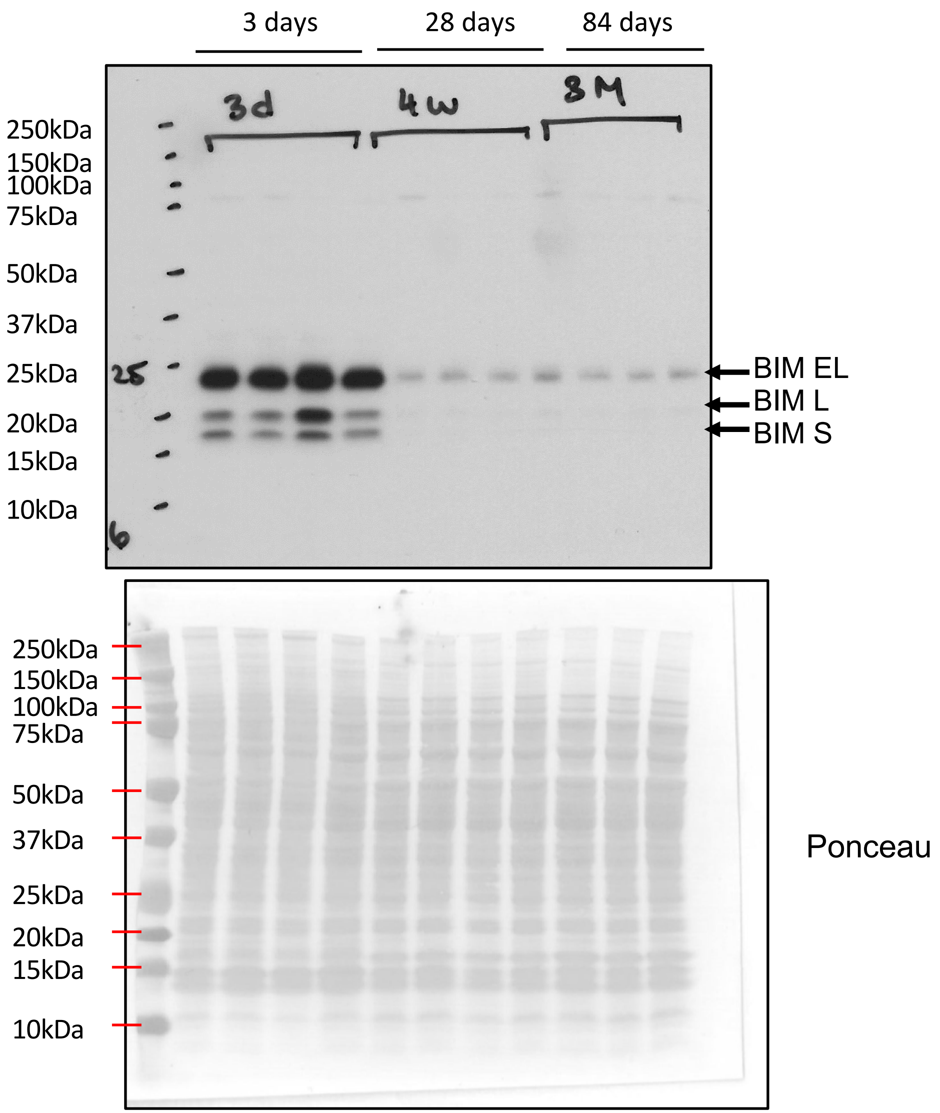

Supplemental Figure 3F

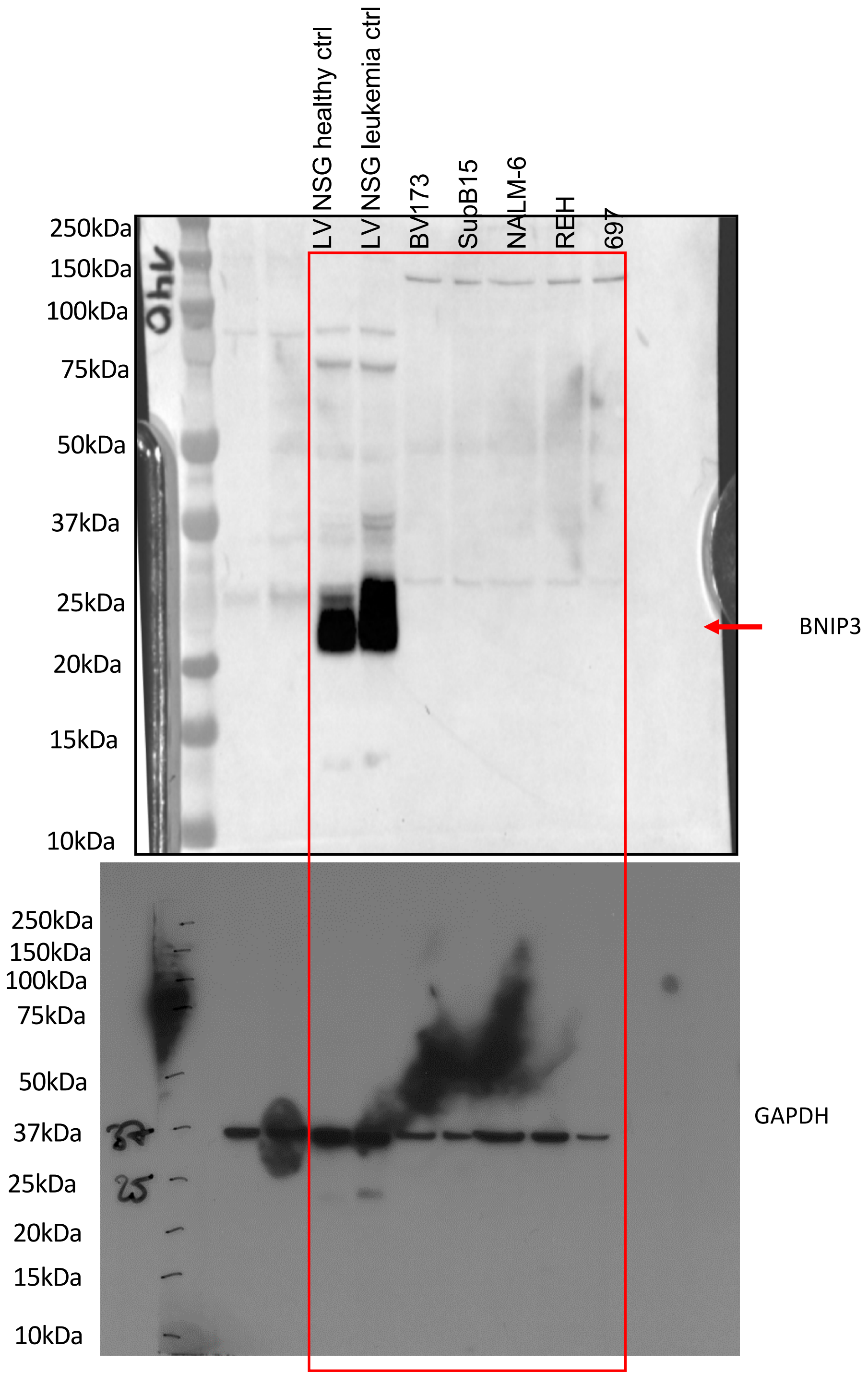

Supplemental Figure S3G

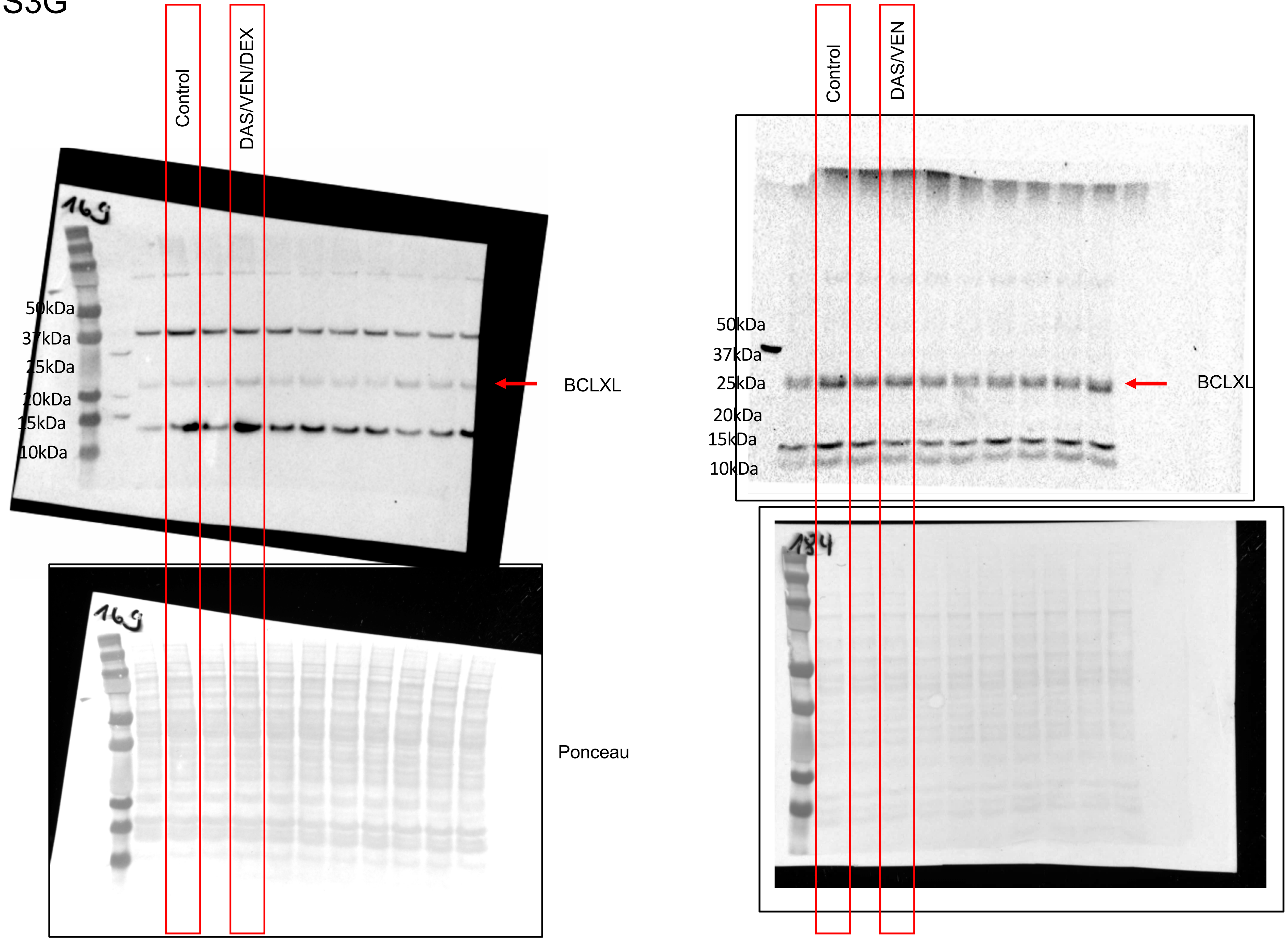

Supplement: Supplementary file 1 [file cancers-14-00983-s001.zip › File S1. Uncropped Western Blots.pdf]
